# Only One Isoform of *Drosophila melanogaster* CTP Synthase Forms the Cytoophidium

**DOI:** 10.1371/journal.pgen.1003256

**Published:** 2013-02-14

**Authors:** Ghows Azzam, Ji-Long Liu

**Affiliations:** Medical Research Council Functional Genomics Unit, Department of Physiology, Anatomy and Genetics, University of Oxford, Oxford, United Kingdom; Princeton University, United States of America

## Abstract

CTP synthase is an essential enzyme that plays a key role in energy metabolism. Several independent studies have demonstrated that CTP synthase can form an evolutionarily conserved subcellular structure termed cytoophidium. In budding yeast, there are two isoforms of CTP synthase and both isoforms localize in cytoophidium. However, little is known about the distribution of CTP synthase isoforms in *Drosophila melanogaster*. Here, we report that three transcripts generated at the CTP synthase gene locus exhibit different expression profiles, and three isoforms encoded by this gene locus show a distinct subcellular distribution. While isoform A localizes in the nucleus, isoform B distributes diffusely in the cytoplasm, and only isoform C forms the cytoophidium. In the two isoform C-specific mutants, cytoophidia disappear in the germline cells. Although isoform A does not localize to the cytoophidium, a mutation disrupting mostly isoform A expression results in the disassembly of cytoophidia. Overexpression of isoform C can induce the growth of the cytoophidium in a cell-autonomous manner. Ectopic expression of the cytoophidium-forming isoform does not cause any defect in the embryos. In addition, we identify that a small segment at the amino terminus of isoform C is necessary but not sufficient for cytoophidium formation. Finally, we demonstrate that an excess of the synthetase domain of CTP synthase disrupts cytoophidium formation. Thus, the study of multiple isoforms of CTP synthase in *Drosophila* provides a good opportunity to dissect the biogenesis and function of the cytoophidum in a genetically tractable organism.

## Author Summary

DNA and RNA are made up from basic building blocks called nucleotides. Those nucleotides also play essential roles in many other biological processes. To separate biological processes within a cell is an important feature of all cell types. For example, mitochondria are specialized structures that contain ATP synthase, the enzyme that makes the nucleotide ATP. While mitochondria and ATP synthase have been studied for about 100 years, it was only very recently that we realized that there are specialized subcellular structures that contain CTP synthase, the enzyme that makes up another basic nucleotide CTP. Several independent studies have shown that CTP synthase molecules can form a filamentous structure called the cytoophidium (meaning “cellular snake” in Greek) or CTP synthase filament in bacteria, budding yeasts, fruit flies, and rat and human cells. In budding yeast, there are two isoforms of CTP synthase and both isoforms localize in the cytoophidium. Here, we report that three CTP synthase isoforms in fruit flies show a distinct subcellular distribution and only one isoform forms the cytoophidium. Thus, the study of multiple isoforms of CTP synthase in the fruit fly gives us a good way to begin to learn how and why CTP synthase molecules form this snake-like structure.

## Introduction

Nucleotides not only serve as building blocks to make up DNA and RNA, but also play critical roles in many additional biological processes. For example, ATP acts as the most widely used biological energy carrier, GTP participates in intracellular signaling and is used as an energy reservoir, and CTP is involved in phospholipid and sialoglycoprotein synthesis. Compartmentation is important for the efficiency of biological processes in cells [1], [2]. Mitochondria and chloroplasts are specialized organelles that contain ATP synthase, the enzyme that makes ATP. The mitochondrion serves as the main site for ATP synthase to generate ATP, and has been considered the ‘power house’ of a cell because it is responsible for producing 90% of the cellular energy. Defects in mitochondria have been linked to a wide range of human diseases such as mitochondrial myopathy, Leigh syndrome, Parkinson's disease and diabetes [3], [4]. Thus, it is not surprising that ATP synthase and the mitochondrion have been extensively studied [5]. By contrast, until very recently, little was known about the subcellular distribution of CTP synthase.

CTP can be synthesized through either the salvage pathway or the *de novo* pathway in many cells [6], [7], [8], [9]. The rate-limiting step of *de novo* CTP biosynthesis is catalyzed by the CTP synthase (CTPsyn) enzyme [6], [7], [8], [9]. CTPsyn catalyses a set of three reactions: a kinase reaction being Mg^2+^-ATP-dependent phosphorylation of the UTP uracil O4 atom; a glutaminase reaction being rate-limiting glutamine hydrolysis to generate ammonia; and a ligase reaction being displacement of the uracil O4 phosphate by ammonia 10,11,12,13,14. In 1978, Weber and co-workers found that CTP synthase activity in hepatomas was elevated [15]. Subsequent studies demonstrated that unregulated CTP levels and increased CTP synthase activity are features of many forms of cancer such as leukemia, hepatomas, and colon cancer [15], [16], [17], [18], [19], 20,21,22,23,24,25,26,27. Furthermore, CTP synthase is an attractive target for drug development against viral [28] and parasitic disease (e.g. African sleeping sickness [29], [30], malaria [31], and infectious blindness [32]).

In 2010, three independent studies reported that CTPsyn is compartmentalized in filamentary structures in bacteria, yeast, fruit flies and rats [33], [34], [35]. More recently, studies have shown that CTPsyn can form filaments in human cells as well [36], [37]. These CTPsyn-containing structures were termed cytoophidia (meaning ‘cellular serpents’ in Greek) [33], [36], CtpS filaments [34], CTP synthase filaments [35], or rods and rings (RR) [37]. For simplicity, the term ‘cytoophidia’ is used in this paper to refer to the apparently equivalent structures that contain CTPsyn (for review see [38]). In addition to CTPsyn, some other metabolic enzymes can form filamentous structures in budding yeast [35] and *Drosophila melanogaster* [39]. Recent studies on the cell biology of CTPsyn suggest that: 1) the cytoophidium is strikingly conserved across prokaryotes and eukaryotes; 2) the cytoophidium is likely to represent a novel type of subcelluar structure; and 3) the conservation of the filament-forming property of CTPsyn offers an exciting opportunity to study the cell biology of metabolic pathways.

Two genes, URA7 and URA8, encode two isoforms of CTPsyn in *Saccharomyces cerevisiae*, and both isoforms have been shown to form filaments [35]. In humans, two isoforms of CTPsyn, which share 74% identity, are encoded by two genes, CTPsyn1 and CTPsyn2. The two human CTPsyn isoforms share 44%–55% identity with the two isoforms of CTPsyn in *S. cerevisiae*. In *Drosophila melanogaster*, CTP synthase is encoded by the gene CG6854. Bioinformatic data suggest that the CG6854 gene locus can produce three transcript variants that correspond to three isoforms. The aim of this study was to determine the expression and subcellular distribution of the individual isoforms, which have so far remained elusive.

Here, we report that three isoforms produced in the CG6854/CTP synthase locus in *D. melanogaster* localize to distinct subcellular compartments, while only one isoform (isoform C) forms the cytoophidium. Overexpression of isoform C can not only increase the length and thickness of cytoophidia in ovarian cells in which cytoophidia are abundant, but also promote cytoophidium assembly in embryos where cytoophidia are less abundant or not detectable. In addition, we identify that a short N-terminal segment that is only present in the cytoophidium-forming isoform is necessary but not sufficient for cytoophidium formation. Finally, we demonstrate that cytoophidium formation can be disrupted by excessive expression of the synthetase domain of CTP synthase. Together, our study of multiple isoforms of CTP synthase in *Drosophila* provides a good opportunity to investigate the biogenesis and function of the cytoophidium in a genetically tractable organism.

## Results

### The CG6854/CTPsyn gene locus encodes three isoforms in *Drosophila melanogaster*

**Figure 1:**
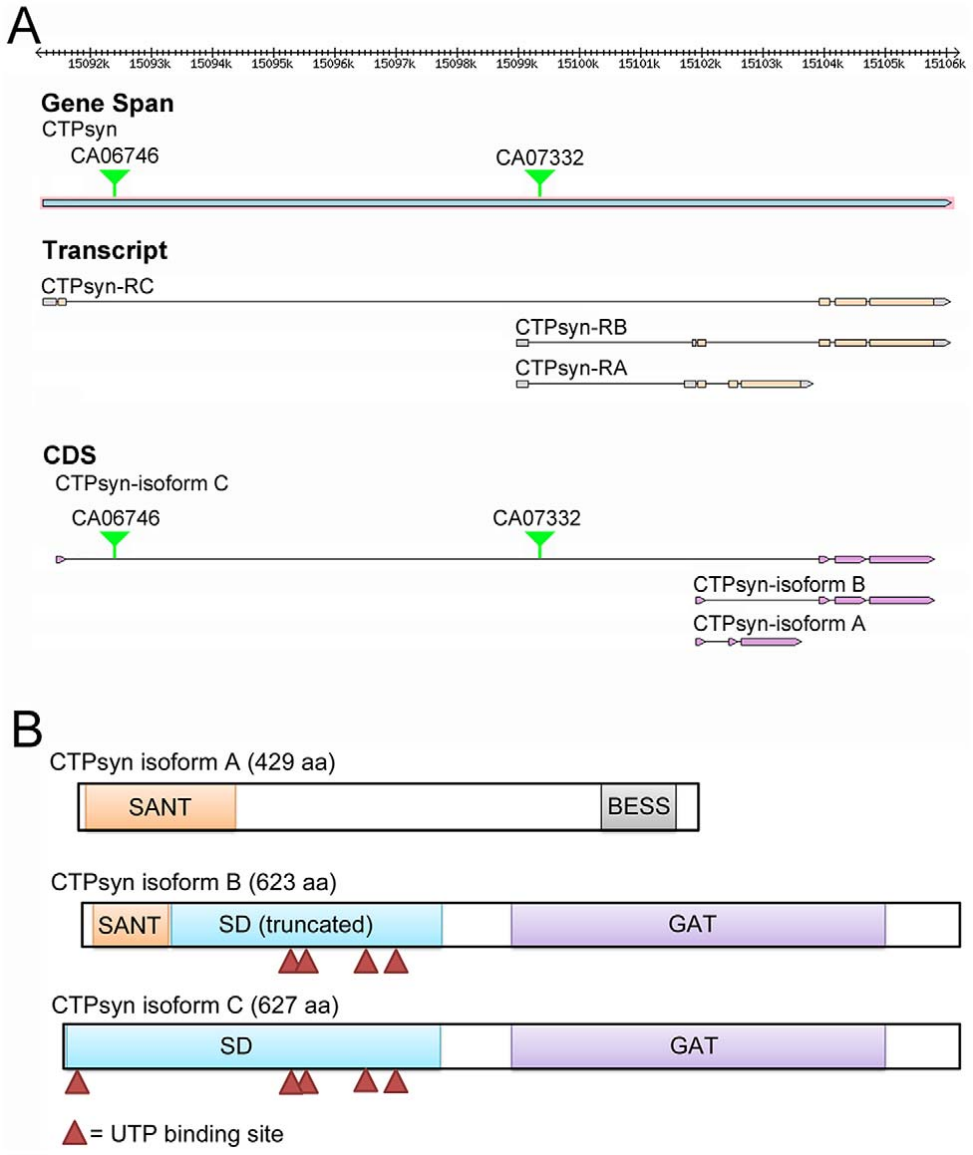
The *Drosophila melanogaster* CG6854/CTPsyn gene locus encodes three isoforms. (A) Genome Browser view of the CG6854/CTPsyn gene locus (www.flybase.org). In two protein trap lines (CA06746 and CA07332), GFP was trapped between the first and second exons of the CTPsyn isoform. (B) The protein map of three isoforms of CTPsyn. UTP binding sites are indicated by red triangles.

In *Drosophila melanogaster*, the *CTPsyn* gene (*CG6854*, *FBgn0262707*) localizes at Chromosome 3L:15091235..15106103 (www.flybase.org). The *Drosophila* CG6854/CTPsyn gene locus produces three transcripts (Figure 1A). The first transcript, which is 1867 nt in length, encodes isoform A, a 429-aa protein. Isoform A consists of two different domains: the N-terminal myb/SANT-like domain in Adf-1 (MADF) and the C-terminal BEAF, Suvar(3)7 and Stonewall (BESS) motif (Figure 1B). This architecture is conserved in at least 14 *Drosophila* proteins [40]. The MADF domain, consisting of 93 aa, is thought to direct the sequence-specific DNA binding to a site consisting of multiple tri-nucleotide repeats. In *D. melanogaster*, 46 genes contain the MADF domain, which is frequently associated with the BESS domain [40], a domain consisting of approximately 40 aa with two predicted alpha helices. The BESS domain, which is predicted to be a DNA binding domain, appears to be specific to *Drosophila* [40].

The second transcript, 2554 nt in length, produced by the CG6854/CTPsyn gene locus encodes the B isoform, a 623-aa protein. The transcript for isoform B contains six exons, among which two exons are shared with the transcript for isoform A. The first 51 aa of isoforms A and B are identical (Figure S1). Isoform B contains two predicted incomplete domains (MADF and CTPsyn synthetase domain) and a type 1 glutamine amidotransferase (GAT) domain (Figure 1A, 1B). The incomplete MADF domain present in this isoform is only 41 aa long, and the truncated CTPsyn synthetase domain consists of 224 aa.

The third transcript of the CG6854/CTPsyn, which is 2406 nt long, produces a 627-aa protein, the isoform C. It contains two domains: the synthetase domain and the GAT domain (Figure 1A, 1B). Isoform C has 5 UTP binding sites that are required for the synthesis of CTP from UTP, while isoform B only has 4 UTP binding sites (Figure 1B).This suggests that only isoform C has the full function of a typical CTPsyn. Orthologous proteins present in organisms from yeast to mammals are mostly aligned to CTPsyn isoform C in *Drosophila* (Figure S2). It appears that two protein trap lines (CA06746 and CA07332) described previously [33], [36], [41] have green fluorescence protein (GFP) trapped in the first and second exons of CTPsyn isoform C (Figure 1B).

### Three transcripts from the CG6854/CTPsyn gene locus exhibit distinct expression profiles

**Figure 2:**
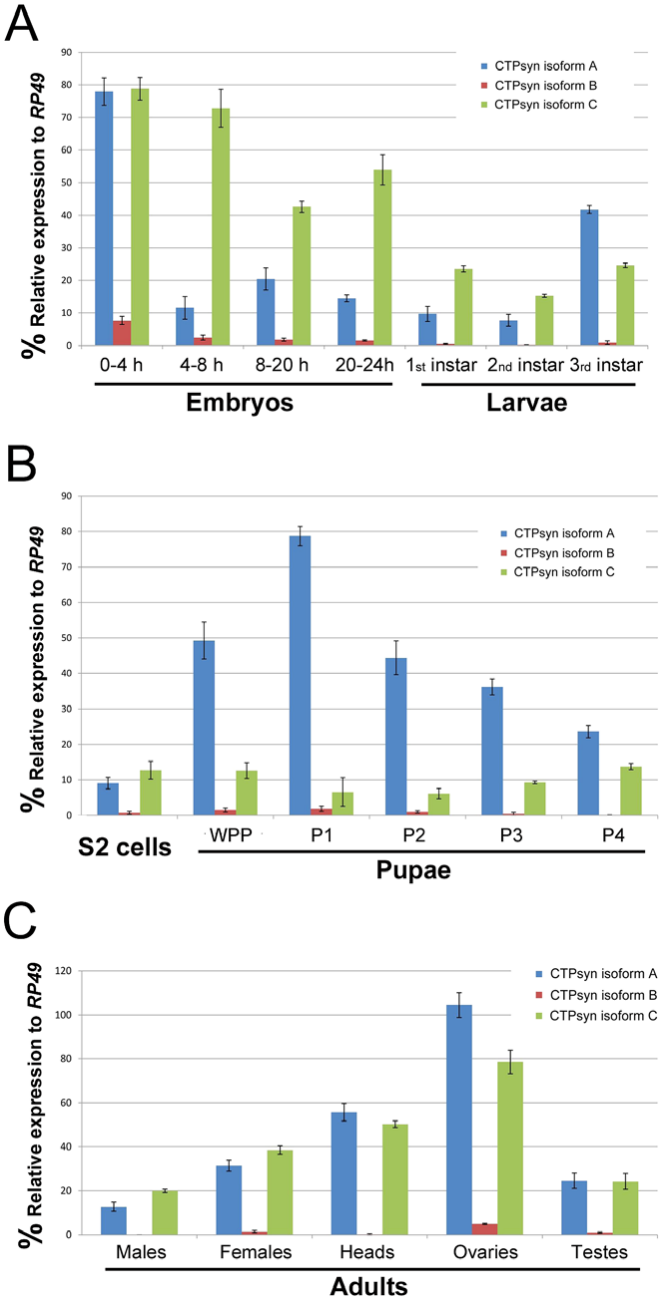
Expression profiles of *Drosophila* CTPsyn isoforms as revealed by qPCR. (A) embryos and larvae. (B) S2 cells and pupae. (C) Adult flies and tissues. WPP, white pre-pupae. P1–P4, pupal stages 1–4.

To determine the expression pattern of the transcripts that encode these three isoforms we performed quantitative PCR (qPCR) using isoform-specific primers (Table S1). We found that the transcript for isoform B was expressed at a very low level in most developmental stages except early embryos (0–4 h) (Figure 2). In contrast, the isoform A and C transcripts were both shown to be very abundant throughout all developmental stages (Figure 2). However, we observed that isoform A and C have very distinct expression profiles. During embryogenesis, both transcripts showed the highest levels in the 0–4 h embryos. However, in embryos at 4–8 h, while the isoform C transcript stayed at a similar level to that in 0–4 h embryos, the transcript for isoform A decreased about 8-fold. In late-stage embryos, the expression of the isoform C transcript decreased 1.5 to 2-fold, while isoform A showed similar levels to those in 4–8 h embyos (Figure 2A). The isoform C transcript showed modest expression in larval and pupal stages, while the isoform A transcript showed very strong expression in third instar larvae that reached a peak in the early pupal stage (Figure 2B). In adult flies, very little expression of isoform B could be detected, while isoforms A and C showed similar and abundant expression, especially in heads and gonads, with the highest level in the ovary (Figure 2C).

### Ectopic expression of isoform C, but not isoform A or B, of CTPsyn affects cytoophidium formation in female germline cells

**Figure 3:**
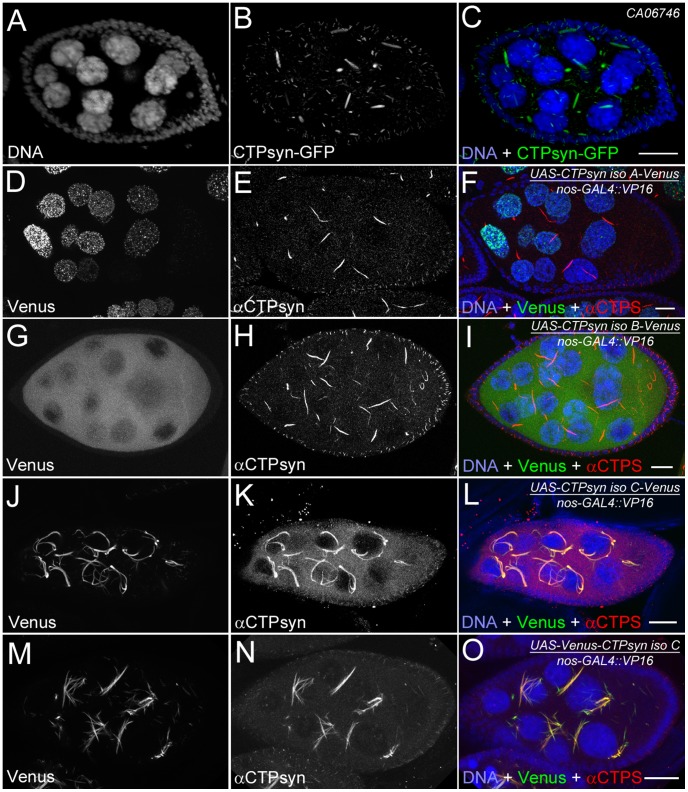
Overexpression of different CTPsyn isoforms in germline cells using nos-GAL4::VP16 driver. (A–C) Cytoophidia revealed by CTPsyn-GFP in an egg chamber from a protein trap line CA06746. (D–F) CTPsyn isoform A is localized in punctate structures in the nucleus and does not affect endogenous cytoophidia. (G–I) CTPsyn isoform B is dispersed in the cytoplasm of the egg chamber and does not affect endogenous cytoophidia. (J–L) C-terminal-tagged CTPsyn isoform C induced longer and more curved cytoophidia. (M–O) N-terminal-tagged CTPsyn isoform C affects cytoophidia morphology. Iso, Isoform. Scale bars, 20 µm.

Our previous studies using multiple antibodies against CTPsyn and two independent CTPsyn-GFP protein trap lines demonstrated that CTPsyn localizes to the cytoophidium in many tissues in *Drosophila* (Figure 3A–3C) [33], [36], [41]. However, it is unclear whether all three isoforms produced by the *Drosophila* CTPsyn gene locus localize to cytoophidia. To determine the distribution of individual isoforms, we made both tagged and untagged constructs of each isoform.

Isoform A, when tagged by Venus either at the N-terminus or C-terminus, localized to punctate structures in the nucleus (Figure 3D–3F). Ubiquitous expression of isoform A transgene by an actin-GAL4 driver resulted in lethality in *Drosophila* at a very early stage. Expression of isoform A transgene in the germline led to small ovaries and sterility in female flies (data not shown). However, cytoophidia, as revealed by immunostaining using antibodies against CTPsyn, did not show any obvious morphological change in germline cells when the isoform A transgene was expressed (Figure 3D–3F).

The CTPsyn isoform B transgene, either with an N-terminal tag or a C-terminal tag, showed a dispersed distribution in the cytoplasm (Figure 3G–3I). The morphology of cytoophidia, as analyzed by antibody staining against CTPsyn, remained unchanged in nurse cells when the isoform B transgene was overexpressed with actin-GAL4. However, compared to flies overexpressing isoform A, overexpressing isoform B in the germline did not cause sterility. Flies overexpressing CTPsyn isoform B ubiquitously were also viable.

The CTPsyn isoform C transgene, when tagged by Venus either at its C-terminus or N-terminus, localized to cytoophidia (Figure 3J–3O). The Venus signal showed an almost identical pattern to that labeled by antibodies against CTPsyn (Figure 3J–3O, Figures S3 and S4). When CTPsyn isoform C with a C-terminus-tagged GFP was overexpressed in female germline cells, we observed that the number and length of macro-cytoophidia increased dramatically (Figure 3J–3L, Figure S3). Many long cytoophidia also appeared very thick. Overexpressing CTPsyn isoform C without a tag showed a similar pattern as that with a C-terminus-tagged Venus (Figure S5). Flies overexpressing isoform C ubiquitously were still viable and fertile, in contrast to those flies overexpressing isoform A. When Venus was tagged to the N-terminus of isoform C, cytoophidia appeared to be straight. The number of cytoophidia increased considerably; the length and thickness of each individual cytoophidium however did not change dramatically (Figure 3M–3O). These short and straight cytoophidia very often tangled up with each other, making them appear spiky (Figure S4). In this case, the Venus tag may obstruct a critical cytoophidium-forming region at the N-terminus of CTPsyn isoform C, as described below. 

### Mutations disrupting isoform C or isoform A expression results in cytoophidium disassembly

**Figure 4:**
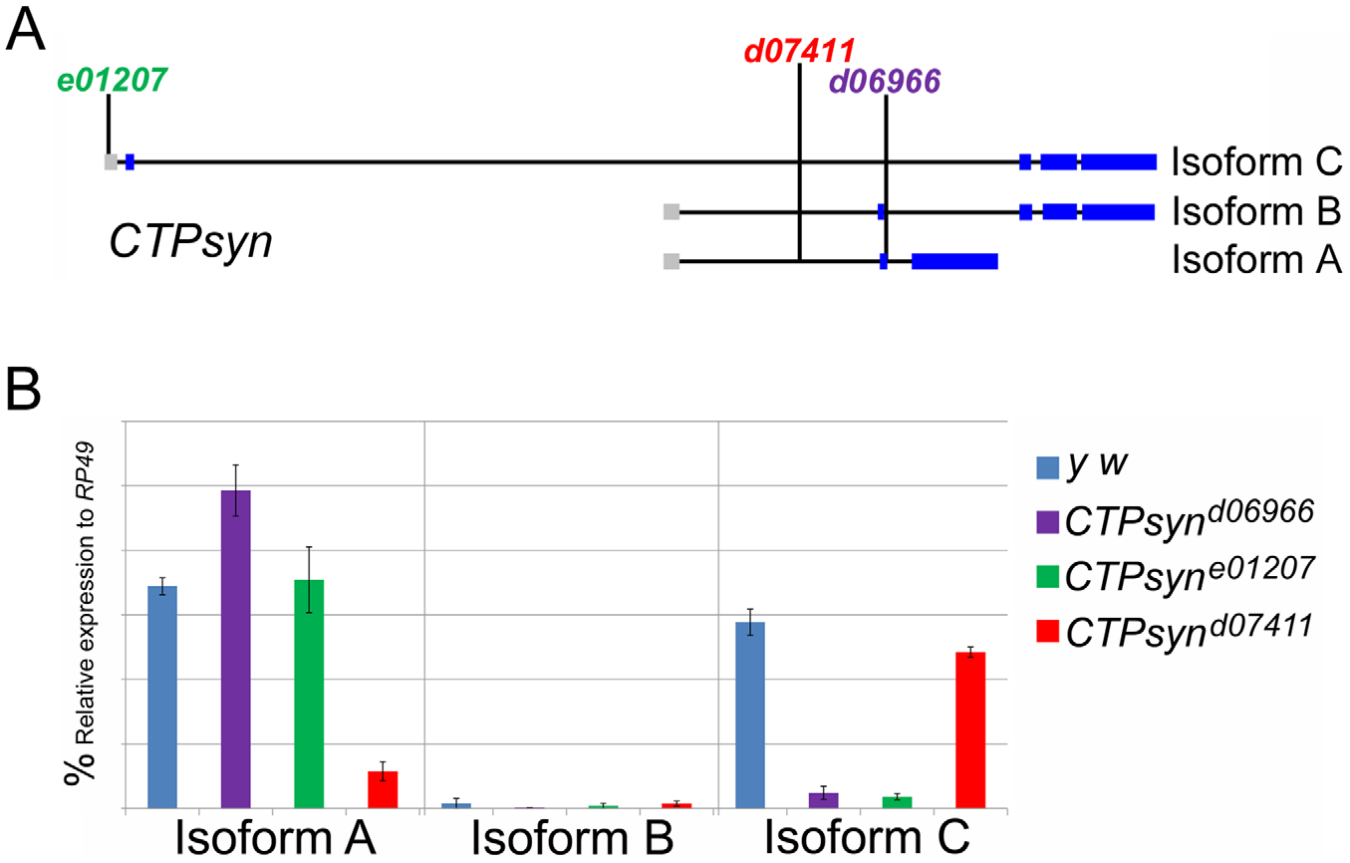
CTPsyn mutants. (A) Genetic map of CTPsyn and the insertion site of *p-element* in three mutants *CTPsyn^d6099^*, *CTPsyn^e01207^* and *CTPsyn^d07411^*. (B) Comparison of the expressions of CTPsyn isoforms in larvae from *y w* and three *CTPsyn* mutants (*CTPsyn^d6099^*, *CTPsyn^e01207^* and *CTPsyn^d07411^*). While dramatic decrease of isofrom C expression occurs in *CTPsyn^d6099^* and *CTPsyn^e01207^*, isoform A expression is hugely diminished in *CTPsyn^d07411^*.

Previous transposon screens yielded a number of inserts in and around the *CTPsyn* locus. We found that three mutants *CTPsyn^d06966^*, *CTPsyn^e01207^* and *CTPsyn^d07411^* (Figure 4A) were homozygous lethal. Complementation analysis showed that *CTPsyn^d06966^* and *CTPsyn^e01207^* failed to complement each other. However, *CTPsyn^d07411^* were able to complement to either *CTPsyn^d06966^* or *CTPsyn^e01207^*, suggesting that *CTPsyn^d07411^* affects different isoform from the other mutants. This is confirmed by qPCR analysis. In comparison with wild-type flies, *CTPsyn^d07411^* flies showed a 6-fold decreased level of the transcript for CTPsyn isoform A, while no change in isoform B and only 20% decrease in isoform C expression (Figure 4B). On the contrary, isoform C expression decreases significantly in both *CTPsyn^d06966^* and *CTPsyn^e01207^* flies (Figure 4B).

**Figure 5:**
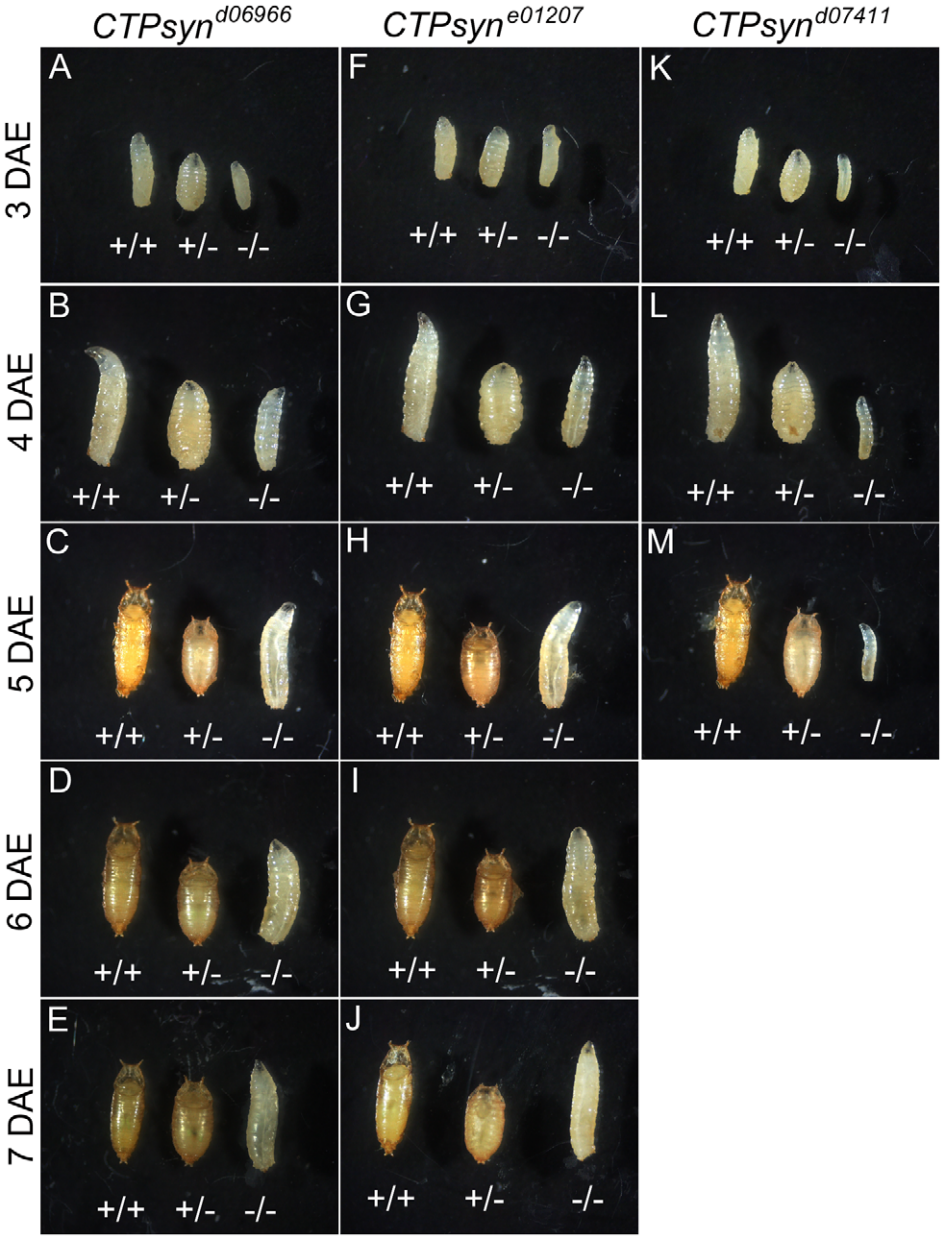
CTPsyn mutants survive until larval stages. *CTPsyn^d6099^* homozygous mutants (−/− in A–E) and *CTPsyn^e01207^* homozygous mutants (−/− in F–J) survive until 7 days after egg deposition (DAE) with slower growth compared to heterozygous mutants (+/−) or y w (+/+). (K–M) *CTPsyn^d07411^* homozygous mutants (−/−) show severely delayed development, in comparison with heterozygous mutants (+/−and *y w* (+/+). *CTPsyn^d07411^* (−/−) mutant larvae only survive until 5 days after egg deposition (DAE) with very little growth. Heterozygous mutants were balanced with TM6B Tb (+/− in all panels).

We balanced these mutants with TM6B which carries the dominant recessive mutation Tubby (Tb) that makes the larvae and pupae have short bodies which are easily distinguishable [42]. Eggs from all three CTPS mutants were collected in an apple juice plates analysed every day from 3 days after egg deposition. From early on, all the mutant larvae were significantly smaller compared to wild-type control (Figure 5). Both *CTPsyn^d06966^* (Figure 5A–5E) and *CTPsyn^e01270^* (Figure 5F–5J) survive and continue to grow until 7 days after egg deposition. However, *CTPsyn^d07411^* only survives until 5 days after egg deposition with very little growth (Figure 5K–5M). Even at 5 days after egg deposition, the *CTPsyn^d07411^* mutant larvae are still very small. All three *CTPsyn* mutants failed to develop into proper pupation stage, although they formed pseudo-pupa occasionally.

**Figure 6:**
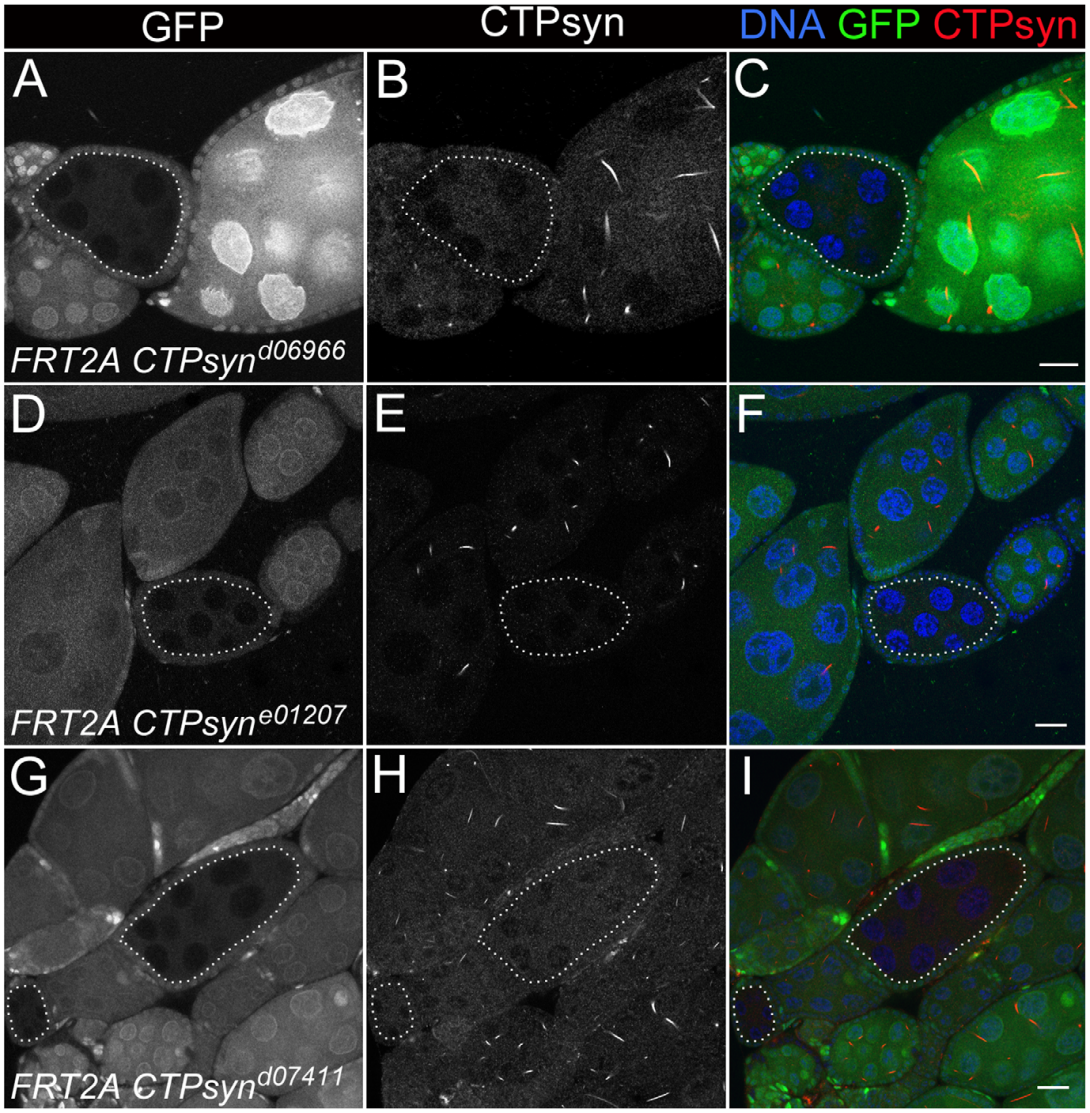
Clonal analysis of three CTPsyn mutations in adult ovaries. (A–C) *CTPsyn^d6099^*. (D–F) *CTPsyn^e01207^*. (G–I) *CTPsyn^d07411^* In all three mutations, no cytoophidia are detected in mutant mitotic clone (dotted lines) as indicated by lack of GFP, while cytoophidia (labelled by an antibody against CTPsyn in red) are clearly seen in adjacent wild-type germline cells (GFP positive). Scale bar, 20 µm.

To study the effect on cytoophidia formation, mitotic clones were generated in female germline cells. As expected, egg chamber clones from two isoform C-specific mutants (*CTPsyn^d06966^* and *CTPsyn^e01207^*) showed disruption in the formation of cytoophidia (Figure 6A–6F). To our surprise, we were unable to detect cytoophidia in the germline cells even from the third mutation *CTPsyn^d07411^* in which isoform A expression was disrupted, while clear cytoophidia could be observed in adjacent wild-type egg chambers (Figure 6G–6I).

### Overexpression of CTPsyn isoform C dramatically increases the length of cytoophidia in follicle cells

In addition to 15 nurse cells and one oocyte, an egg chamber contains several hundred to a thousand follicle cells, which form a monolayer epithelium surrounding the large nurse cells and the oocyte [43]. Our previous studies indicate that each follicle cell contains only one cytoophidium. Cytoophidia exhibit a similar length in follicle cells within the same egg chamber [33]. This unique feature of cytoophidia makes the follicle cell epithelium an ideal model to study the biogenesis of the cytoophidium.

**Figure 7:**
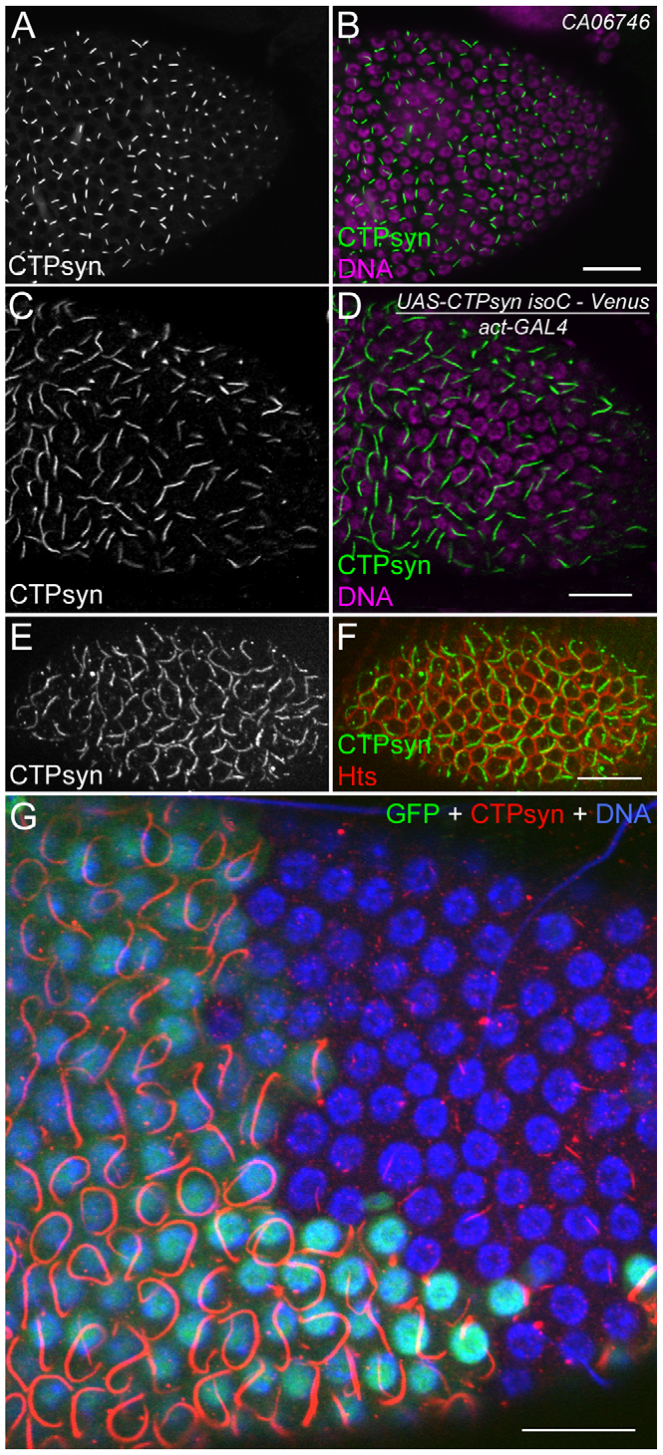
Overexpression of CTPsyn isoform C induces the assembly of cytoophidia in *Drosophila* follicle cells. (A, B) Cytoophidia revealed by CTPsyn-GFP in protein trap line CA06746. (C, D) Long cytoophidia are induced in stage-10 follicle cells overexpressing CTPsyn isoform C. (E, F) Each follicle cell contains only one long cytoophidium in a stage-7 egg chamber. The cell boundary is outlined by membrane protein Hu-li tai shao (Hts). (G) Within the same stage 10 egg chamber, cytoophidia in follicle cells overexpressing CTPsyn isoform C (GFP+ cells) are much longer and thicker than those in neighbouring wild-type follicle cells (GFP− cells). While the average length of cytoophidia in GFP− cells is only 3.97±0.61 µm (n = 41), cytoophidia in GFP+ cells are 20.76±5.60 µm long on average (n = 32). Note that cytoophidia in GFP+ cells in E are much longer than those in C and D, which could be due to different expression of GAL4 drivers in follicle cells (actin-GAL4 in C, D and tubulin-GAL4 in E). Scale bars, 20 µm.

To better understand the role of CTPsyn isoform C in cytoophidium formation, we analyzed cytoophidia in follicle cells from flies in which this isoform was overexpressed. We predicted several possible outcomes: the number of cytoophidia could increase from one to many, the length of the cytoophidia could increase, or both changes could happen. Our results showed that the length of cytoophidia increased dramatically in the follicle cells (Figure 7A–7D). In CTPsyn protein trap CA06746 flies, cytoophidia are less than 2 µm in early-stage follicle cells (average 1.92±0.23 µm at stages 4 to 6, n = 54) and they reach average 3.23±0.47 µm at stage 9 (n = 196) and 3.81±0.47 µm at stage 10A (n = 80). When the expression of CTPsyn isoform C transgene was driven by actin-GAL4, we observed that the average lengths of cytoophidia can reach to 5.30±0.77 µm at stage 6 (n = 36), 9.33±1.54 µm at stage 9 (n = 69), and 9.77±1.36 µm at stage 10A (n = 54), respectively (Figure 7A–7D). However, in many cases, there was still only one cytoophidium per follicle cell even when overexpressing isoform C (Figure 7E, 7F).

To test if CTPsyn isoform C cell-autonomously affects cytoophidium formation, we generated mitotic clones in which nuclear GFP labeled a patch of cells overexpressing isoform C. We observed that cytoophidia in cloned cells were much thicker and longer than in the neighboring wild-type cells (Figure 7G). Follicle cells within a clone contained cytoophidia of similar lengths. The extended cytoophidia curled up around the nucleus in many cells, as they were presumably restrained by the cytoplasm. Wild-type follicle cells near the clones had cytoophidia of similar lengths to those cells that were further away from the clones, suggesting that the level of CTPsyn isoform C regulates the formation of cytoophidia in a cell-autonomous manner.

### Embryos are tolerant to highly abundant cytoophidia

**Figure 8:**
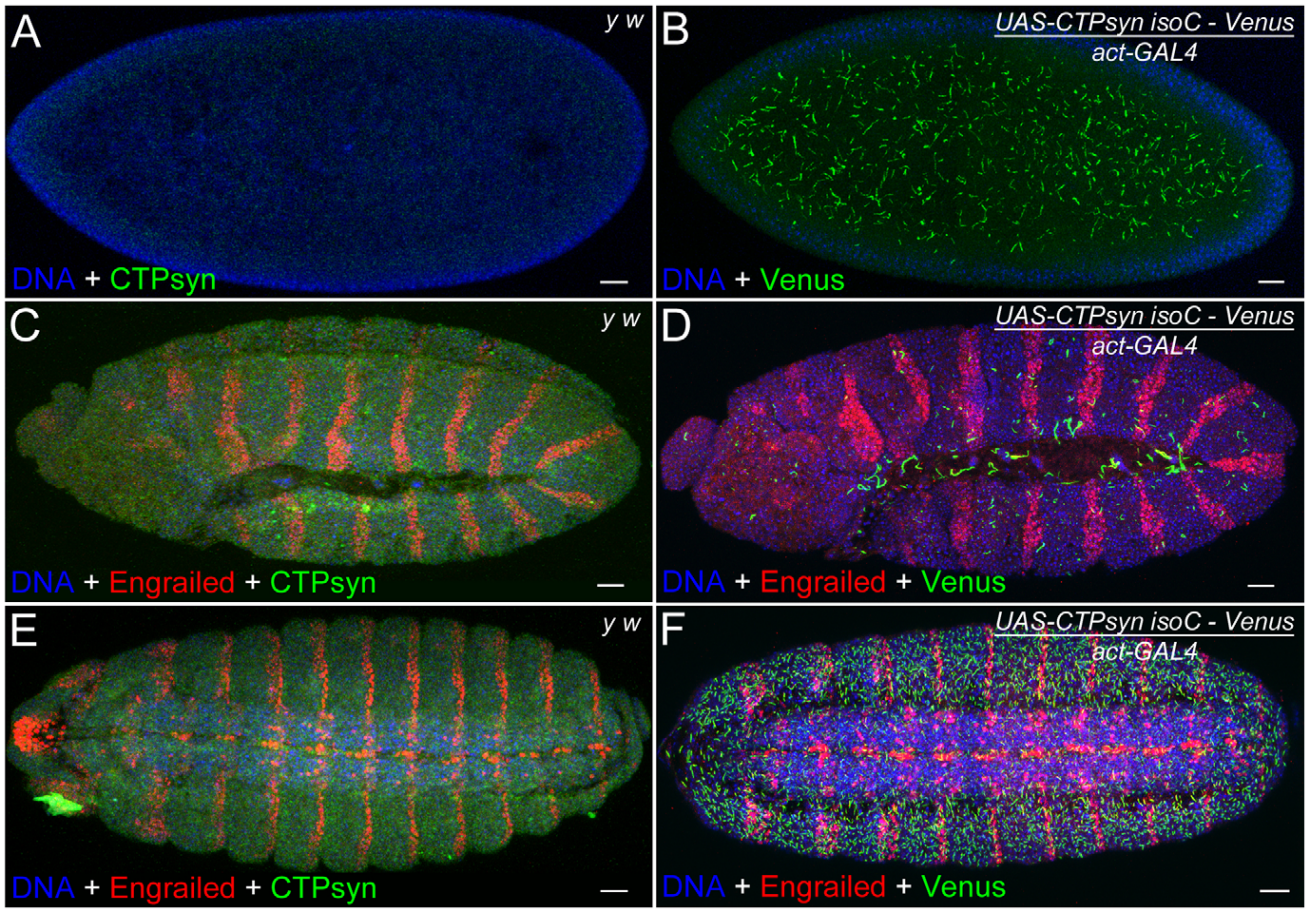
Overexpression of CTPsyn isoform C induces macro-cytoophidia in *Drosophila* embryos. (A, C, E) No macro-cytoophidium formation can be detected in *y w* embryos at stage 1 (A), 12 (C) and 15 (E). (B, D, F) Overexpressing CTPsyn isoform C induces macro-cytoophidia in embryos at stage 1 (B), 12 (D), and 15 (F). Segments in embryos are labeled by a transcription factor Engrailed (red). IsoC, isoform C. Scale bars, 20 µm.

The above results in the ovary suggest that CTPsyn isoform C can dramatically affect the length of cytoophidia in cells that normally contain cytoophidia. Previous studies indicate that not every cell contains a detectable cytoophidium [33], [35], [36]. We have observed that *Drosophila* embryos do not exhibit detectable large cytoophidia (i.e. macro-cytoophidia), either by immunostaining with antibodies against CTPsyn or by CTP syn::GFP protein trap line (Figure 8A, 8C, 8E). To determine if CTPsyn isoform C can induce *de novo* cytoophidium assembly, we ectopically expressed this isoform in *Drosophila* embryos. Our results showed that in embryos overexpressing CTPsyn isoform C, cytoophidium formation was promoted throughout embryogenesis, starting from stage 1 (Figure 8B, 8D, 8F). We observed more cytoophidia in stage 15 embryos than in stage 12 embryos (Figure 8F), which could be due to differential expression of the actin-GAL4 driver during embryogenesis. Embryos with highly abundant cytoophidia developed normally. These results indicate that CTPsyn isoform C plays a critical role in the *de novo* assembly of cytoophidia, which seemingly do not impair embryogenesis in *Drosophila*.

### N-terminal region of isoform C is necessary but not sufficient for cytoophidium formation

**Figure 9:**
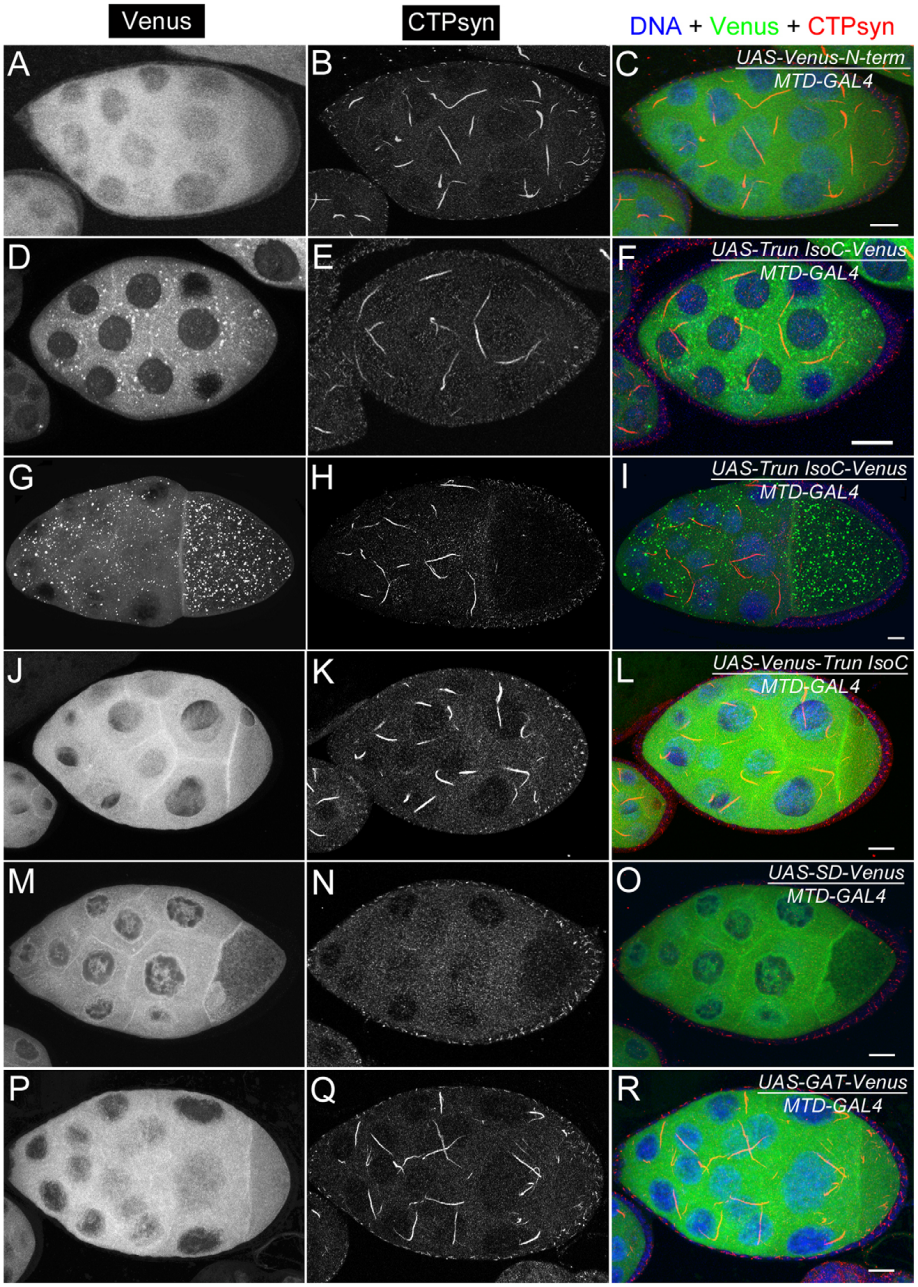
Ectopic expression of transgenes in egg chambers using *MTD-GAL4* driver. (A–C) UAS-Venus-N-term which consists of the 56 amino acids shows dispersed cytoplasmic expression. (D–I) UAS-Trun isoC-Venus forms punctate structures in the cytoplasm. The punctate structures are more visible in egg chambers at later stages (G–I) than in those at early stages (D–F). (J–L) UAS-Venus-Trun isoC shows dispersed distribution in the cytoplasm. (M–O) Ectopic expression of UAS-SD-Venus disrupts endogenous cytoophidium formation in the germline cells. Note that there is no expression of UAS-SD-Venus in follicle cells in which cytoophida are not affected. (P–R) UAS-GAT-Venus shows disperse distribution in the cytoplasm. See Figure S6 for the structures of the constructs. N-term, a N-terminal segment (56-aa) of CTPsyn isoform C; Trun IsoC, truncated isoform C. SD, synthetase domain; GAT, type 1 glutamine amidotransferase domain. Scale bars, 20 µm.

Most regions of CTPsyn isoform B are identical to those of isoform C. The only difference between these two isoforms lies in their amino (N) terminus: 56 amino acids in isoform C and 52 amino acids in isoform B (Figure 1A, 1B). To identify the critical regions for cytoophidium formation, we generated transgenic flies that carried Venus-tagged constructs based on various regions of CTPsyn isoform C (the cytoophidium-forming isoform) (Figure S6). Each of these transgenes were induced by a maternal triple driver (MTD) so their cytoophidium-forming ability could be monitored in the female germlines (Figure 9).

We found that a truncated isoform C without a 56-aa N-terminal segment (N-term) was unable to form cytoophidia, suggesting that N-term plays an important role in cytoophidium formation (Figure 9D–9L). As shown above, we noted that Venus tagging at the N-terminal of full-length isoform C affected the morphology of cytoophidia. This may be because the Venus tag interferes with this critical cytoophidium-forming segment. To test if N-term alone is sufficient for cytoophidium formation, we tagged Venus with N-term and found that N-term-GFP did not localize to the cytoophidum (Figure 9A–9C). Our results indicate that N-term of CTPsyn isoform C is necessary but not sufficient for the formation of cytoophidia.

### Excess of CTPsyn synthetase domain disrupts cytoophidium formation

The concentration of CTPsyn affects the equilibrium between its monomeric, dimeric and tetrameric forms [44]. Each monomer contains two functional domains: the synthetase domain and the GAT domain [45], [46], [47]. The GAT domain catalyses GTP-activated glutamine hydrolysis, while the synthetase domain mediates Mg^2+^-ATP-dependent phosphorylation of the UTP uracil O4 atom and displacement of the uracil O4 phosphate by ammonia [48], [49]. CTPsyn activity requires oligomerization, and each synthetase active site and essential ATP- and UTP-binding surfaces are contributed by three monomers [13]. The triphosphate moiety of the CTP product overlaps the binding site for the UTP substrate, while the CTP cytosine ring resides at a separate site [14].

Using time-lapse microscopy, Gitai and co-workers have shown that mCherry-CTPsyn can grow from a focus to a long filament in curved bacterium *Caulobacter crescentus* [34]. In the same study, they have also shown that purified *E. coli* CTPsyn molecules can form filaments *in vitro* [34]. Therefore, it is likely that CTPsyn in a cytoophidiun is in its polymeric form. Overexpression of isoform C induces cytoophidium formation, suggesting that the concentration of CTPsyn also affects the equilibrium between its oligomeric and polymeric forms. Since the synthetase domain is critical for oligomerization of CTPsyn, we hypothesized that an excess of the synthetase domain, by competitively binding full-length CTPsyn to form oligomers, can disrupt the polymerization of CTPsyn to form cytoophidia.

To test this hypothesis, we generated a synthetase domain transgene tagged with Venus. When this transgene was overexpressed in female germline cells, we found that the Venus signal was dispersed in the cytoplasm and did not localize to the cytoophidium (Figure 9M–9O). As predicted, the endogenous CTPsyn failed to form detectable cytoophidia in germline cells while cytoophidia in follicle cells were not affected. Consistent with this idea, we found that overexpression of the GAT domain, which would be dispensable for tetramerization of CTPsyn, showed no obvious effect on endogenous cytoophidium formation (Figure 9P–9R).

**Figure 10:**
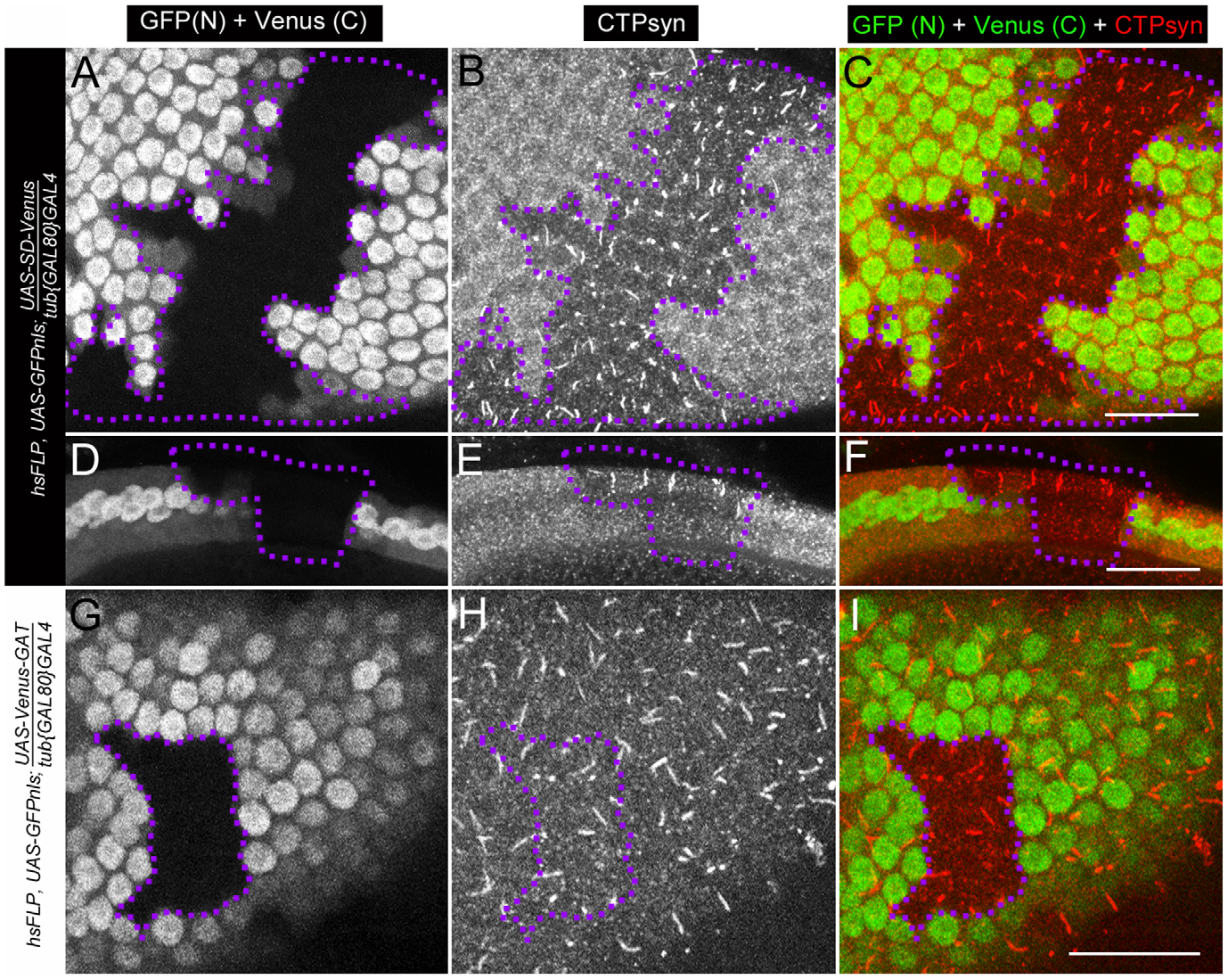
Clonal expression of CTPsyn domains in follicle cells using inducible driver. (A–F) Ectopic expression of UAS-SD-Venus disrupts endogenous cytoophidium formation in follicle cells (GFP+ cells), while cytoophidia are detectable in wild-type follicle cells (GFP− cells, within dotlined-area). (A–C) Surface view. (D–F) Lateral view. (G–I) Ectopic expression of UAS-Venus-GAT has no obvious effect on cytoophidium formation in follicle cells. Note that cytoophidia are detectable in both GFP+ (where the transgene is expressed) and GFP− cells (dotlined, wild-type). SD, synthetase domain; GAT, type 1 glutamine amidotransferase domain. Venus (C), cytoplasmic signal from Venus. GFP (N), nuclear signal from GFP. Scale bars, 20 µm.

To better understand the effect of ectopic expression of these two domains, we analysed cytoophidia in follicle cells. Using inducible driver, we generated mitotic clones in which nuclear GFP labeled those cells expressing the transgene of interest. In our case, the transgenes were synthetase domain or GAT domain, both of which were tagged with Venus. When follicle cells contained excess synthease domain-Venus, cytoophidia were not longer maintained, while wild-type follicle cells in the same egg chamber have obvious cytoophidia (Figure 10A–10F). Similar to the results obtained from germline cells, overexpression Venus-GAT domain did not show obvious effect on cytoophidium formation in follicle cells (Figure 10G–10I).

## Discussion

### Are there three isoforms or three genes at the *Drosophila* CTPsyn gene locus?

Three transcripts generated at the CTPsyn gene locus give rise to three protein products in *Drosophila melanogaster* (CTPsyn-PA, PB and PC, Figure 1A which distribute differently within the cell. Do the three protein products belong to three isoforms of the same protein or should they be considered as products from different genes? The arguments that suggest that the three products are derived from different genes as follows: CTPsyn-PA and CTPsyn-PC do not share any amino acid sequence, and they have very distinct localizations, with PA in the nucleus and PC in cytoplasmic cytoophidia. While orthologous proteins of CTPsyn-PC are much conserved from bacteria to human, CTPsyn-PA and PB seem to be unique to *Drosophila* (Figure S2). Among those three protein products only CTPsyn-PA contains the BESS domain, which appears to be specific to *Drosophila*. The BESS domain can bind to DNA, which may explain the nuclear localization of CTPsyn-PA. Thus it appears reasonable to suggest that the *Drosophila* CTPsyn gene locus contains three genes, with only one encoding full-length CTPsyn, of which cytoophidium are composed.

However, there are several lines of evidence to support those three protein products being considered as isoforms derived from the same gene. First, three transcripts from the CTPsyn gene locus in *Drosophila melanogaster* are overlapping, not only at untranslated regions, but also at coding sequences. Second, at the protein level, although CTPsyn-PA and PC are non-overlapping, they both overlap with CTPsyn-PB at different regions. It appears that CTPsyn-PB is the bridge that links PA and PC at the same locus. Third, even though PA, PB and PC have distinct subcellular localizations, there are examples of different isoforms of the same protein showing distinct localizations in the cell. Chan and colleagues [37] have shown that another nucleotide metabolic enzyme, IMP dehydrogenase (IMPDH), colocalizes with CTPsyn in the rods and rings (i.e. cytoophidia) in human and mouse cell lines. A more recent study in *Drosophila* suggests that IMPDH can localize both in the cytoplasm as a metabolic enzyme and in the nucleus as a transcriptional factor [50]. Finally, in the *CTPsyn^d07411^* mutant, the formation of cytoophidia is disrupted. Since the transcript of *CTPsyn-PC* only has 20% decrease in *CTPsyn^d07411^* (in comparison, the transcript of *CTPsyn-PA* decreases its expression by 83%), it is unlikely that the disassembly of cytoophidia in *CTPsyn^d07411^* could be explained by the change of CTPsyn-PC alone. A possible explanation is that cytoophidia not only requires CTPsyn-PC, but also requires CTPsyn-PA for assembly, even though the latter does not localize on the cytoophidium. This suggests that there is a functional link between CTPsyn-PA and PC in *Drosophila*. Further analysis of multiple alleles at the CTPsyn gene locus in *Drosophila* will shed new insights into the relationship of these three isoforms.

### Requirements for cytoophidium assembly

There are an abundant of cytoophidia in female germline cells, i.e nurse cells and oocytes, especially during mid-oogenesis. There are also two types of cytoophidia in nurse cells and oocytes: macro- and micro-cytoophidia. The numbers of macro-cytoophidia are less at late stage oogenesis, suggesting that the assembly and disassembly of cytoophidia is linked to developmental status. Overexpressing CTPsyn isoform C can not only promote cytoophidium assembly in cells with abundant cytoophidia, such as nurse cells, oocytes and follicle cells in the ovaries, but also induce the formation of cytoophidia in tissues that usually have few, if any, macro-cytoophidia. This suggests that CTPsyn isoform C is a major factor promoting cytoophidium assembly.

CTPsyn isoform A is distributed in the nucleus, yet a mutation primarily disrupting isoform A affects cytoophidium formation in the cytoplasm. How does a nuclear protein affects a structure in the cytoplasm? One possibility is that CTPsyn isoform A might affect isoform C post-transcriptionally. Another possible explanation could be that the biogenesis of cytoophidia requires a phase in the nucleus, which somehow is dependent on isoform A. Alternatively, the maintenance of cytoophidia might require one or a few factors which are regulated by isoform A. Further detailed studies of isoform A are necessary to get insight into the assembly of cytoophidia.

The sequence of CTPsyn isoform B is 91% identical with that of isoform C, yet only the latter forms the cytoophidium. It suggests that the 56-aa present in the N-terminus of CTPsyn isoform C but not in isoform B is critical for the formation of cytoophidia, as confirmed experimentally in this study. This N-terminal region is highly conserved among CTPsyn molecules from bacteria to humans; thus our results in *Drosophila* would most likely be acceptable across species. When these 56 aa were tagged with Venus at its N-terminus we did not observe cytoophidium localization. Two possibilities can be considered. One possible explanation is that the large Venus tag (about 260 aa) interferes with cytoophidium formation sites at this short peptide (56 aa). It is also possible that the formation of cytoophidia requires multiple binding sites including the ones presenting at those N-terminal 56 aa.

Overexpressing the synthetase domain of CTPsyn isoform C disrupts the formation of endogenous cytoophidia. The excessive synthetase domain might competitively bind and block some critical cytoophidium-forming sites that present in the full-length CTPsyn isoform C. Those critical cytoophidium-forming sites could be uncoupled from catalytic sites in CTPsyn isoform C. Gitai and colleagues have shown that a point mutation at the synthetase domain (G147A) of *Caulobacter crescentus* CTPsyn that inactive a catalytic site does not cause any detectable change in the frequency or morphology of filamentous structure [34].

In summary, the assembly of cytoophidia appears to be a multiple-step process. While CTPsyn isoform A is primarily localizing in the nucleus, a mutation specific to this nuclear isoform disrupts the formation of cytoophidia in the cytoplasm. Further studies on the relationship of different CTPsyn isoforms in *Drosophila* would be helpful to understand the biogenesis of the cytoophidium.

Note added in proof: While this paper was under review, a new release from the Flybase (www.flybase.org; FB2012_06, released November 6th, 2012) showed a fourth transcript of at the CG6854/CTPsyn gene locus, which is 2062 nt in length, encoding CTPsyn isoform D (429 aa). While the CTPsyn isoform D protein has the exact sequence as isoform A, the first exon of the CTPsyn isoform D transcript overlaps with the first and second exons of the CTPsyn isoform C transcript.

## Materials and Methods

### Fly stocks

All stocks were raised at 25°C on standard cornmeal media. *y w* flies were used as a control in all experiments if not indicated. CTP synthase GFP protein trap lines, CA06746 and CA07332, were gifts from Michael Buszczak and Allan Spradling [41]. The inducible GAL4 driver stock *hsFLP*, *UAS-GFPnls*; *tub-{GAL80}-GAL4* was used to generate mosaic overexpression and *hsFLP*, *UAS-GFPnls*; *UAS-Dicer-2*; *tub-{GAL80}-GAL4* was used to generate mosaic overexpression of transgenes [51]. *CTPsyn* mutant stocks *(CTPsyn^e01207^ (PBac{RB})*, *CTPsyn^d06966^ (P{XP})*, *CTPsyn^d07411^ (P{XP}) used in this study* were obtained from Bloomington stock centre and the Harvard Exelixis collection [52].

### Total RNA extraction and reverse transcription

RNA extraction was performed by homogenizing samples using the Qiagen QIAshredder (Cat. no. 79654) and RNA was extracted using the Qiagen RNeasy Plus Mini Kit (Cat. No. 74134) as per the manufacturer's instructions. Samples were kept at −80°C. Reverse transcription was carried out using the Qiagen QuantiTect Rev. Transcription Kit (Cat. no. 205311) with the gDNA removal step following the manufacturer's instructions. The cDNA were then further diluted 1∶10 with nuclease-free water and kept at −20°C.

### Quantitative PCR (qPCR)

About 1 µl of diluted cDNA from the reverse transcription was mixed with Fast SYBR Green Master Mix (Applied Biosystems Cat. no. 4385612) and 1 µM of primers (Table S1) for each 10 µl qPCR reactions. The reactions were carried out using the 7500 Fast Real-Time PCR System (Applied Biosystems) on the Fast setting: initial denaturation at 95°C for 20 s, denaturation at 95°C for 3 s, primer annealing and elongation at 60°C for 30 s, repeated for 40 cycles, then final denaturation at 95°C for 15 s, and final primer annealing and elongation at 60°C for 1 min. Expression values were normalized using reference gene *RP49*.

### Transgenic flies

To make the untagged constructs, the gene sequence was amplified by PCR from cDNA using Pfx polymerase (Invitrogen 11708-021) and specific primers with additional restriction site sequences (Table S2). Both PCR products and the pUASp-K10-attB vector were then digested individually using specific restriction enzymes and ligated using T4 DNA ligase (NEB M202). The product was then sequenced before being injected into embryos.

All tagged constructs were made using an enhanced GFP or Venus [53] tagged UASp vector from the Gateway clones (Terrance Murphy collection), and the untagged constructs were made using the modified pUASp vector which contains attB sites for PhiC31 integrase-mediated site-specific injection [54]. To make the constructs, these cDNA clones were used: LD27370 (isoform A), LD27537 (isoform B) and LP25005 (isoform C), which were acquired from the *Drosophila* Genomics Resource Center gold collections.

To make the Venus tagged construct, the gene sequence was amplified by PCR from specific cDNA using Pfx polymerase (Invitrogen 11708-021) and isoform-specific primers (Table S3) that have additional sequences as described by the *Drosophila* Gateway Vector Collection protocol. The PCR products were then cloned into a pENTR using the pENTR/D-TOPO cloning kit (Invitrogen K240020). Then, to make the destination construct, the sequences cloned into the pENTR vector were recombined into the UASp-Venus (N-terminal tagged or C-terminal tagged) vector using LR clonase II (Invitrogen 11791020). The final constructs were then sequenced before being sent to be injected.

Embryo injections were carried out by GenetiVision Inc. (Texas, USA). Constructs with the attB sequence were injected using the PhiC31 integrase-mediated site-specific technique with specific landing site [55]. P-element constructs were injected and the progenies were scored using eye marker.

### Inducible GAL4 system

To generate clones overexpressing CTPsyn transgenes, *hsFLP*, *UAS-GFPnls*; *sp*; *tub {GAL80} GAL4/SM5*, *Cy-TM6 Tb* flies were crossed to *UASp-CTPsyn isoform C*. To generate follicle cell clones, eggs were collected in vials for 24 h and heat-shocked after 4 days in a 37°C water bath for 1 h. The cells expressing the construct were marked by the presence of GFP in the nucleus.

### Mitotic clones in female germlines

Three CTPsyn mutants (*CTPsyn^d06966^*, *CTPsyn^e01207^* and *CTPsyn^d07411^*) were used in this study. A FRT site located at the 79D-F region (FRT2A) was recombined to all three lines. Mitotic clones were generated by crossing *y w*; *CTPsyn^mutant^ e FRT2A/TM3 Ser* males to *hsFLP; ubi GFP FRT2A/*TM3-Ser females. The resulting progeny were heat-shocked at 37°C for 1 h during the third instar larval stages. Ovaries from the female progeny of genotype *y w*/hsFLP ; *CTPsyn^mutant^* e FRT2A/Ubi-GFP FRT2A were harvested and stained for GFP. Egg chambers homozygous for the mutation were negative for GFP in the germline, whereas wild-type egg chambers were marked with GFP.

### Immunochemistry

*Drosophila* ovaries were dissected in Grace's insect medium (Invitrogen Cat. no. 11605045) and fixed with 4% paraformaldehyde for 10 min, washed with PBT (PBS+0.4% Triton X-100), blocked with 5% horse serum for 1 h and incubated in primary antibodies at room temperature overnight. Samples were washed with PBT and then incubated overnight with the DNA dye Hoechst 33342 and secondary antibodies. Primary antibodies used in this study included rabbit anti-CTPsyn (1∶1000; y-88, sc-134457, Santa Cruz BioTech Ltd, Santa Cruz, CA, USA), mouse anti-Hu-li tao shao (Hts) (1∶20; 7H9 1B1, Developmental Studies Hybridoma Bank, Iowa City, IA, USA) and mouse anti-Engrailed (1∶1000; 4D9, Developmental Studies Hybridoma Bank). Secondary antibodies used in this study were anti-mouse, rabbit, goat or guinea pig antibodies that were labeled with Alexa Fluor 488, 546 or 633 dyes (Molecular Probes), or with Cy5 or Dylight 649 (Jackson ImmunoResearch Laboratories, Inc.).

### Confocal microscopy

All samples were examined and captured under laser-scanning confocal microscopes (Zeiss LSM 510 META, Oberkochen, Germany; and Leica TCS SP5II, Leica Microsystems CMS GmbH, Mannheim, Germany). The lengths of cytoophidia in follicle cells were measured by tracing with straight or segmented lines using ImageJ (v1.43 U) (http://rsbweb.nih.gov/ij/).

## Supporting Information

Figure S1 Comparison of three transcripts and their products at the CTPsyn gene locus in *Drosophila melanogaster*. CTPsyn isoform B overlaps with isoform A at the N-terminal 51 aa (red box), while isoforms B and C are identical for 571 aa (blue box) apart from their N-termini.
(TIF)

Figure S2 CTPsyn orthologous proteins in different species. *Drosophila* CTPsyn-PC is evolutionarily conserved, while CTPsyn-PA and –PC appear more specific to *Drosophila*.
(TIF)

Figure S3 Expression of CTPsyn isoform C-Venus in female germline cells. Cytoophida are long and curly when overexpressing CTPsyn isoform C tagged with Venus at its C-terminus. (A) DNA. (B) Venus. (C) An antibody against CTPsyn shows almost identical pattern as Venus. (D) Merge of A, B and C. Note that this figure is a zoom-in view of the same egg chamber shown in Figure 3J–3L.
(TIF)

Figure S4 Expression of Venus-CTPsyn isoform C in female germline cells. Cytoophida are relative short and straight when overexpressing CTPsyn isoform C tagged with Venus at its N-terminus. (A) DNA. (B) Venus. (C) An antibody against CTPsyn shows almost identical pattern as Venus. (D) Merge of A, B and C. Note that this figure is a zoom-in view of the same egg chamber shown in Figure 3M–3O.
(TIF)

Figure S5 Overexpressing CTPsyn isoform C without a tag in female germline cells. Overexpression of CTPsyn isoform C without a tag shows similar pattern to that of overexpression of CTPsyn isoform C tagged with Venus at its C-terminus (See Figure S3), but very different from that tagged with Venus at its N-terminus (see Figure S4). (A) DNA. (B) An antibody against CTPsyn. (C) An antibody against Hu-li tai shao (Hts), a membrane protein. (D) Merge of A, B and C.
(TIF)

Figure S6 The domains of CTPsyn isoform C in which the transgenes were generated. Four transgenes, N-term, Truncated Isoform C, Synthetase domain (SD) and type 1 glutamine amidotransferase domain (GAT) domains were generated based on CTPsyn isoform C. N-term, an N-terminal segment (56-aa) of CTPsyn isoform C.
(TIF)

Table S1 Sequences of primers used for quantitative PCR (qPCR).
(DOCX)

Table S2 Sequences of primers used for generating untagged constructs.
(DOCX)

Table S3 Sequences of primers used for generating Venus-tagged constructs.
(DOCX)
