# Supplementary figures and images for: Only One Isoform of Drosophila melanogaster CTP Synthase Forms the Cytoophidium
Source: PLoS Genet. 2013 Feb 14;9(2):e1003256. doi: 10.1371/journal.pgen.1003256 (PMC3573105; doi:10.1371/journal.pgen.1003256)

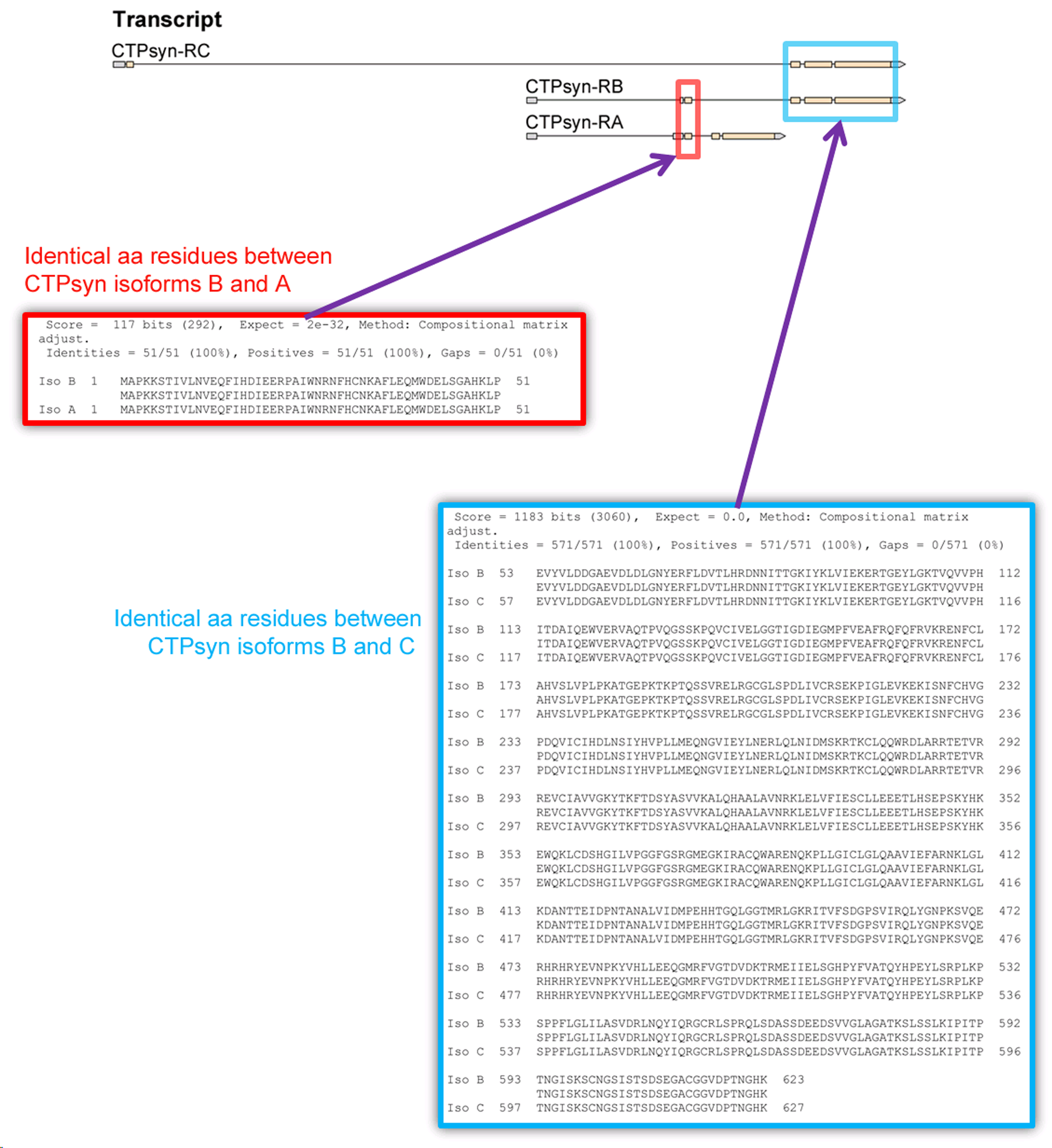

Supplement: Figure S1 — Comparison of three transcripts and their products at the CTPsyn gene locus in Drosophila melanogaster. CTPsyn isoform B overlaps with isoform A at the N-terminal 51 aa (red box), while isoforms B and C are identical for 571 aa (blue box) apart from their N-termini. (TIF) [file pgen.1003256.s001.tif]

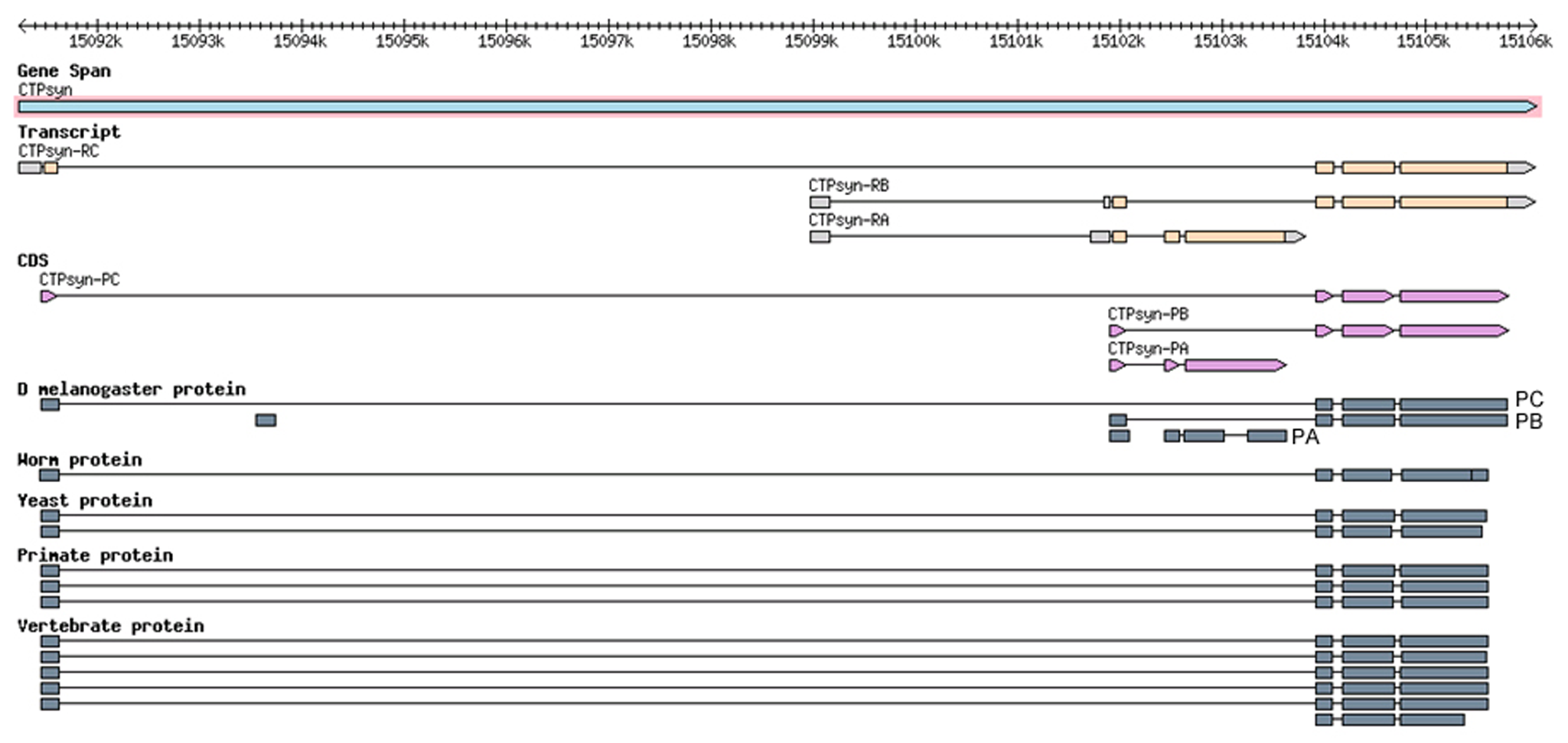

Supplement: Figure S2 — CTPsyn orthologous proteins in different species. Drosophila CTPsyn-PC is evolutionarily conserved, while CTPsyn-PA and –PC appear more specific to Drosophila. (TIF) [file pgen.1003256.s002.tif]

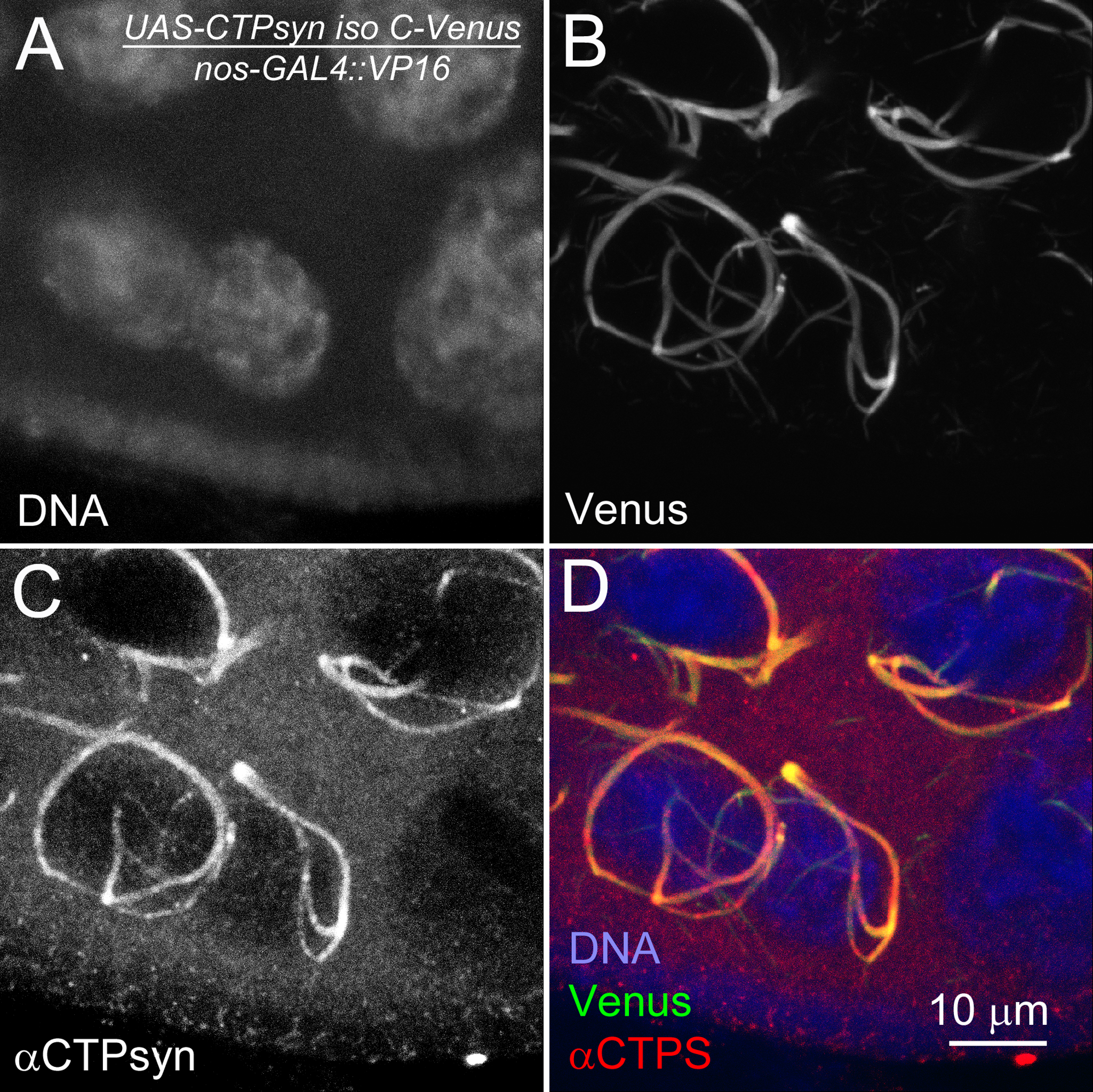

Supplement: Figure S3 — Expression of CTPsyn isoform C-Venus in female germline cells. Cytoophida are long and curly when overexpressing CTPsyn isoform C tagged with Venus at its C-terminus. (A) DNA. (B) Venus. (C) An antibody against CTPsyn shows almost identical pattern as Venus. (D) Merge of A, B and C. Note that this figure is a zoom-in view of the same egg chamber shown in Figure 3J–3L. (TIF) [file pgen.1003256.s003.tif]

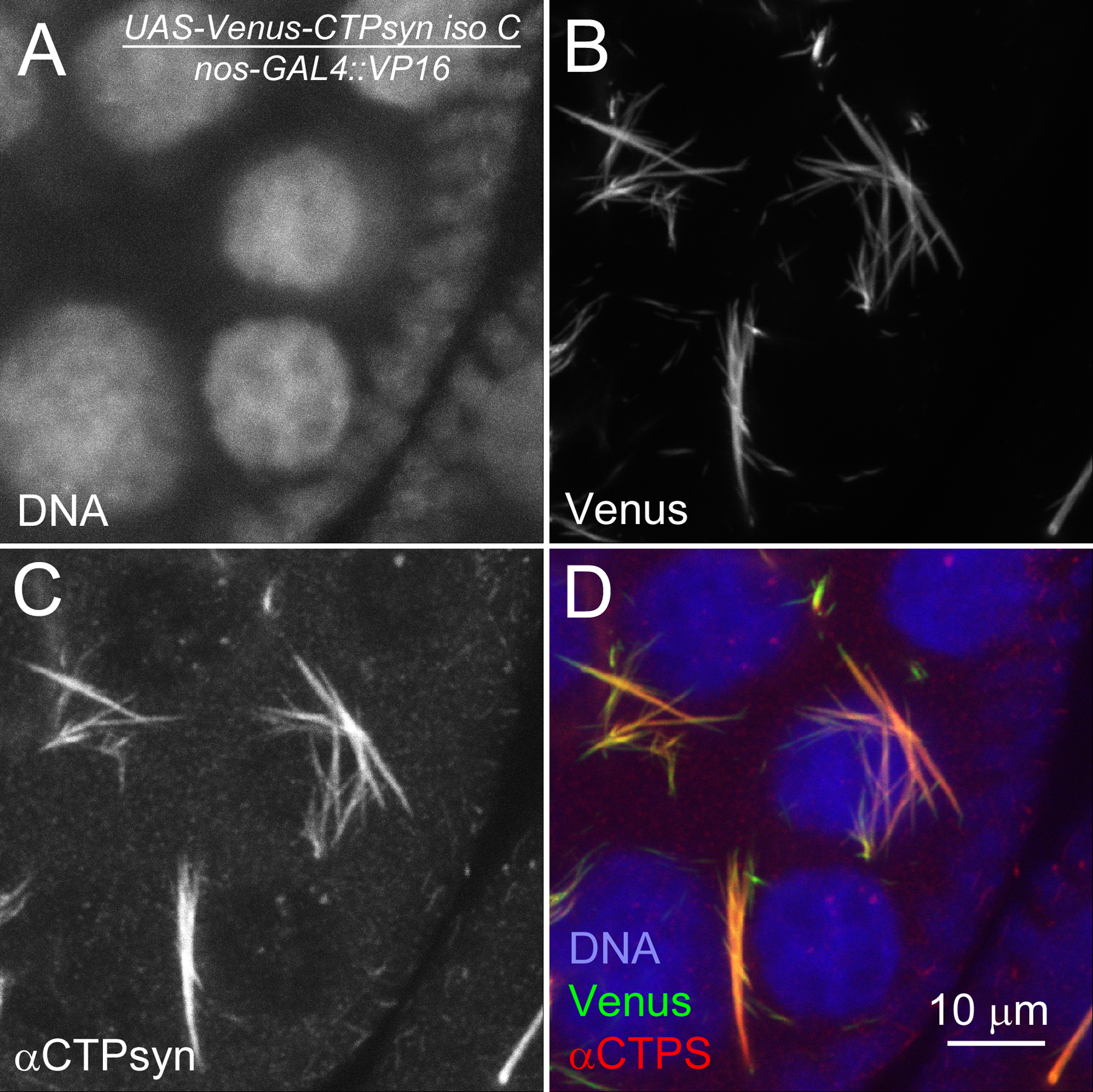

Supplement: Figure S4 — Expression of Venus-CTPsyn isoform C in female germline cells. Cytoophida are relative short and straight when overexpressing CTPsyn isoform C tagged with Venus at its N-terminus. (A) DNA. (B) Venus. (C) An antibody against CTPsyn shows almost identical pattern as Venus. (D) Merge of A, B and C. Note that this figure is a zoom-in view of the same egg chamber shown in Figure 3M–3O. (TIF) [file pgen.1003256.s004.tif]

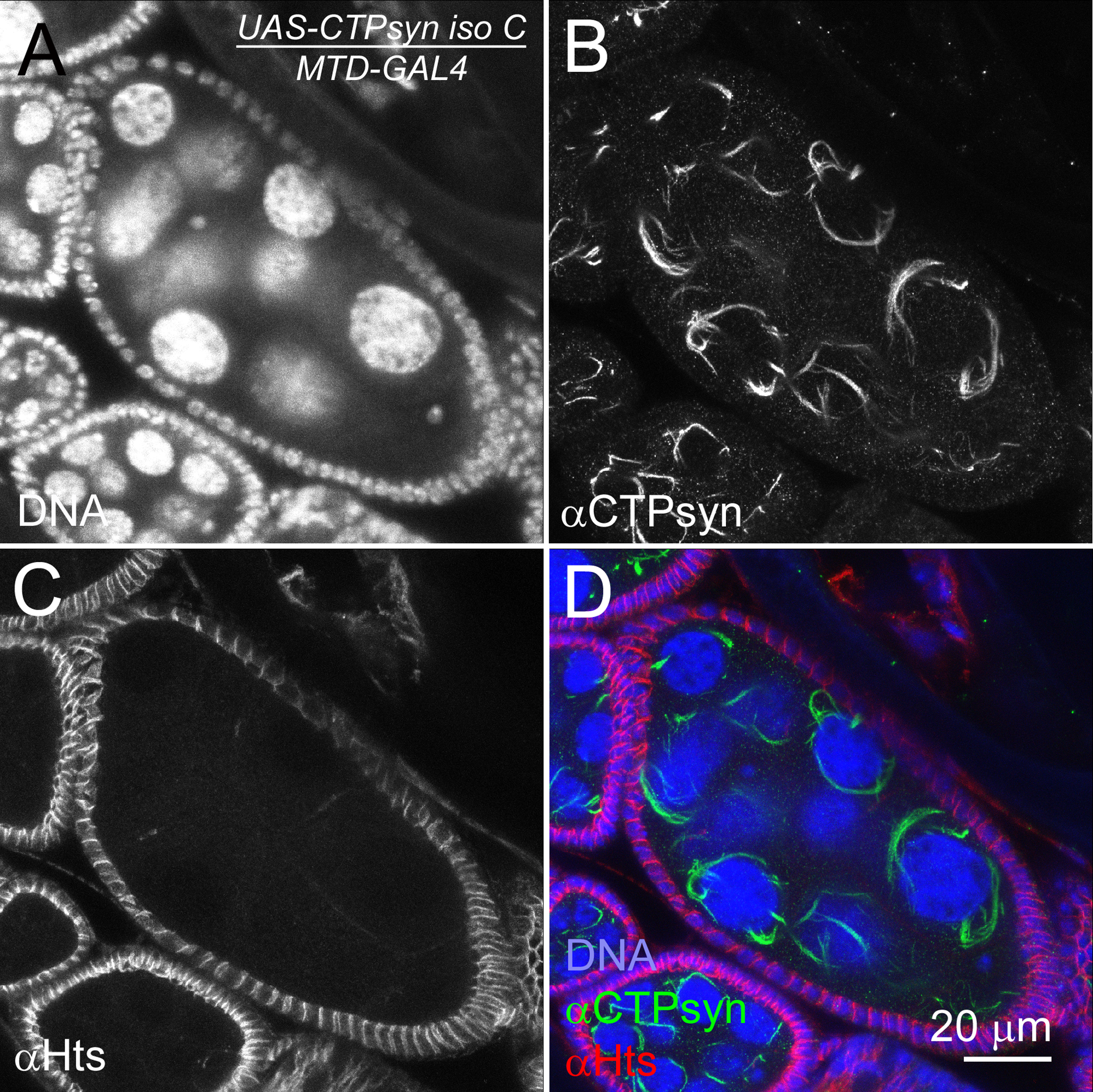

Supplement: Figure S5 — Overexpressing CTPsyn isoform C without a tag in female germline cells. Overexpression of CTPsyn isoform C without a tag shows similar pattern to that of overexpression of CTPsyn isoform C tagged with Venus at its C-terminus (See Figure S3), but very different from that tagged with Venus at its N-terminus (see Figure S4). (A) DNA. (B) An antibody against CTPsyn. (C) An antibody against Hu-li tai shao (Hts), a membrane protein. (D) Merge of A, B and C. (TIF) [file pgen.1003256.s005.tif]

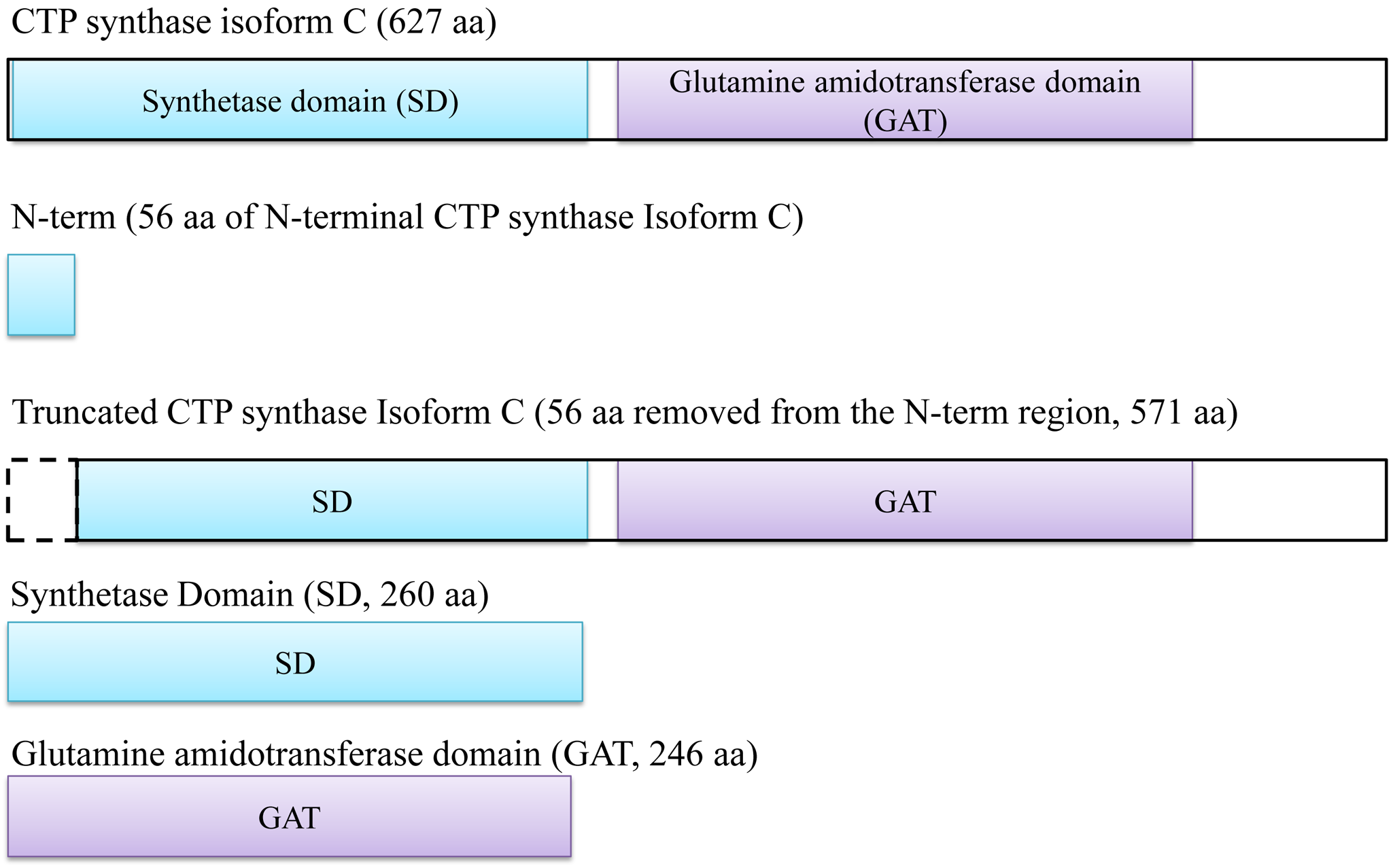

Supplement: Figure S6 — The domains of CTPsyn isoform C in which the transgenes were generated. Four transgenes, N-term, Truncated Isoform C, Synthetase domain (SD) and type 1 glutamine amidotransferase domain (GAT) domains were generated based on CTPsyn isoform C. N-term, an N-terminal segment (56-aa) of CTPsyn isoform C. (TIF) [file pgen.1003256.s006.tif]
