# Supplementary material for: Only One Isoform of Drosophila melanogaster CTP Synthase Forms the Cytoophidium
Source: PLoS Genet. 2013 Feb 14;9(2):e1003256. doi: 10.1371/journal.pgen.1003256 (PMC3573105; doi:10.1371/journal.pgen.1003256)
Supplement: Table S1 — Sequences of primers used for quantitative PCR (qPCR). (DOCX) [file pgen.1003256.s007.docx]

| **Primer Name** | **Sequence** |
| --- | --- |
| CTPsyn isoform A  Forward  Reverse | AGTGGTTCGCATTTTGGTTC  GCACTCACGCACACCACTAT |
| CTPsyn isoform B  Forward  Reverse | ACTCACTCAATCACTTTGAG  GATCCAGATCCACCTCGGCG |
| CTPsyn isoform C  Forward  Reverse | GAGTGATTGCCTCCTCGTTC TCCAAAAACCGTTCATAGTT |
