# Supplementary material for: Only One Isoform of Drosophila melanogaster CTP Synthase Forms the Cytoophidium
Source: PLoS Genet. 2013 Feb 14;9(2):e1003256. doi: 10.1371/journal.pgen.1003256 (PMC3573105; doi:10.1371/journal.pgen.1003256)
Supplement: Table S2 — Sequences of primers used for generating untagged constructs. (DOCX) [file pgen.1003256.s008.docx]

| **Primer Name** | **Sequence** | **Product Size (bp)** |
| --- | --- | --- |
| UASp-CTPsyn isoA  Forward  Reverse | GTGGTACCATGGCGCCAAAAAAGTCCAC  GTTCTAGACTACTTGGACTCCTCAAGAT | 1596 |
| UASp-CTPsyn isoB  Forward  Reverse | GTGGTACCATGGCGCCAAAAAAGTCCAC  GTGGATCCTTACTTATGGCCATTGGTAG | 2423 |
| UASp-CTPsyn isoC  Forward  Reverse | GTGGTACCATGAAATACATCCTGGTAAC  GTGGATCCTTACTTATGGCCATTGGTAG | 2397 |
