# Supplementary material for: Only One Isoform of Drosophila melanogaster CTP Synthase Forms the Cytoophidium
Source: PLoS Genet. 2013 Feb 14;9(2):e1003256. doi: 10.1371/journal.pgen.1003256 (PMC3573105; doi:10.1371/journal.pgen.1003256)
Supplement: Table S3 — Sequences of primers used for generating Venus-tagged constructs. (DOCX) [file pgen.1003256.s009.docx]

| **Primer Name** | **Sequence** | **Product Size (bp)** |
| --- | --- | --- |
| UASp-CTPsyn isoA-Venus  Forward  Reverse | CACCATGGCGCCAAAAAAGTCCAC  CTTGGACTCCTCAAGATCTTCTTTGTAG | 1596 |
| UASp-Venus-CTPsyn isoA  Forward  Reverse | CACCGCGCCAAAAAAGTCCACCAT  CTACTTGGACTCCTCAAGATCTTCTTTGT | 1596 |
| UASp-CTPsyn isoB-Venus  Forward  Reverse | CACCATGGCGCCAAAAAAGTCCAC  CTTATGGCCATTGGTAGGAT | 2423 |
| UASp-Venus-CTPsyn isoB  Forward  Reverse | CACCGCGCCAAAAAAGTCCACCAT  TTACTTATGGCCATTGGTAG | 2423 |
| UASp-CTPsyn isoC-Venus  Forward  Reverse | CACCATGAAATACATCCTGGTAAC  CTTATGGCCATTGGTAGGAT | 2397 |
| UASp-Venus-CTPsyn isoC  Forward  Reverse | CACCAAATACATCCTGGTAACTGGTGGCG  TTACTTATGGCCATTGGTAG | 2397 |
| UASp-N term-Venus  Forward  Reverse | CACCATGAAATACATCCTGGTAAC  CATGCTCATAAGGCGA | 166 |
| UASp-Venus-N term  Forward  Reverse | CACCAAATACATCCTGGTAACTGGTGGCG  TTACATGCTCATAAGGCGA | 169 |
| UASp-Trunc isoC-Venus  Forward  Reverse | CACCATGGAGGTTTACGTTTTGGACG CTTATGGCCATTGGTAGGAT | 1879 |
| UASp-Venus-Trun isoC  Forward  Reverse | CACCGAGGTTTACGTTTTGGACG TTACTTATGGCCATTGGTAG | 1886 |
| UASp-SD-Venus  Forward  Reverse | CACCATGAAATACATCCTGGTAAC  TTCACGGCGAACGGTCT | 895 |
| UASp-Venus-SD  Forward  Reverse | CACCAAATACATCCTGGTAACTGGTGGCG  TTATTCACGGCGAACGG | 898 |
| UASp-GAT-Venus  Forward  Reverse | CACCATGATTGAATACCTAAATGA CTTATGGCCATTGGTAGGAT | 1169 |
| UASp-Venus-GAT  Forward  Reverse | CACCATTGAATACCTAAATGA CATGCTCATAAGGCGA | 1166 |

Abbreviations: N-term, an N-terminal segment (56-aa) of CTPsyn isoform C; Trun IsoC, truncated isoform C; SD, synthetase domain; GAT, type 1 glutamine amidotransferase domain.
